# Laccase-mimicking Mn–Cu hybrid nanoflowers for paper-based visual detection of phenolic neurotransmitters and rapid degradation of dyes

**DOI:** 10.1186/s12951-022-01560-0

**Published:** 2022-08-02

**Authors:** Thao Nguyen Le, Xuan Ai Le, Tai Duc Tran, Kang Jin Lee, Moon Il Kim

**Affiliations:** Department of BioNano Technology, Gachon University, 1342 Seongnamdae-ro, Sujeong-gu, Seongnam, 13120 Gyeonggi Republic of Korea

**Keywords:** Protein-free nanoflower, Laccase-mimicking nanozyme, Colorimetric detection, Paper microfluidic devices, Neurotransmitter detection

## Abstract

**Background:**

Laccase-based biosensors are efficient for detecting phenolic compounds. However, the instability and high cost of laccases have hindered their practical utilization.

**Results:**

In this study, we developed hierarchical manganese dioxide–copper phosphate hybrid nanoflowers (H–Mn–Cu NFs) as excellent laccase-mimicking nanozymes. To synthesize the H–Mn–Cu NFs, manganese dioxide nanoflowers (MnO_2_ NFs) were first synthesized by rapidly reducing potassium permanganate using citric acid. The MnO_2_ NFs were then functionalized with amine groups, followed by incubation with copper sulfate for three days at room temperature to drive the coordination interaction between the amine moieties and copper ions and to induce anisotropic growth of the petals composed of copper phosphate crystals, consequently yielding H–Mn–Cu NFs. Compared with those of free laccase, at the same mass concentration, H–Mn–Cu NFs exhibited lower *K*_*m*_ (~ 85%) and considerably higher *V*_*max*_ (~ 400%), as well as significantly enhanced stability in the ranges of pH, temperature, ionic strength, and incubation periods evaluated. H–Mn–Cu NFs also catalyzed the decolorization of diverse dyes considerably faster than the free laccase. Based on these advantageous features, a paper microfluidic device incorporating H–Mn–Cu NFs was constructed for the convenient visual detection of phenolic neurotransmitters, including dopamine and epinephrine. The device enabled rapid and sensitive quantification of target neurotransmitters using an image acquired using a smartphone.

**Conclusions:**

These results clearly show that H–Mn–Cu NFs could be potential candidates to replace natural laccases for a wide range of applications in biosensing, environmental protection, and biotechnology.

**Graphical Abstract:**

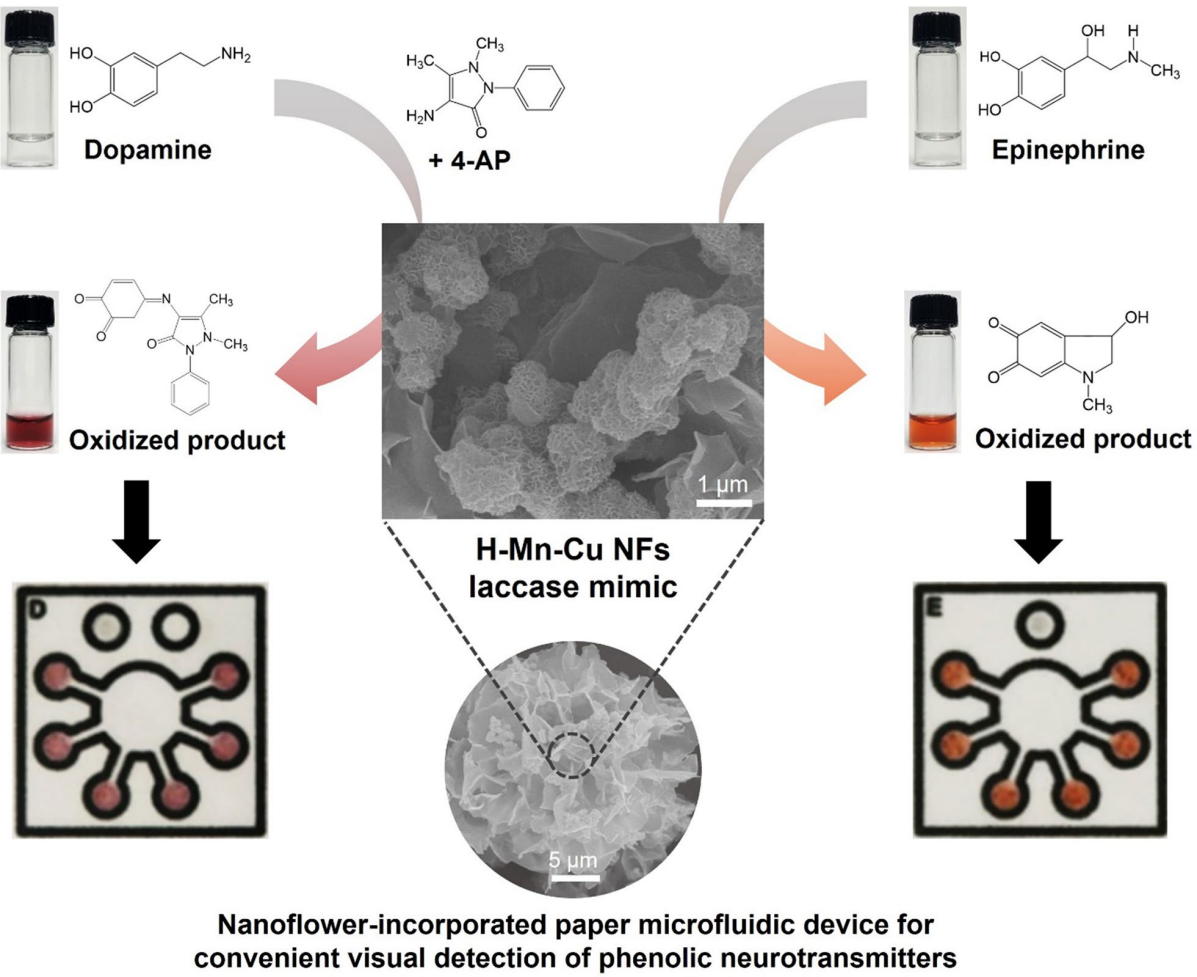

**Supplementary Information:**

The online version contains supplementary material available at 10.1186/s12951-022-01560-0.

## Background

Phenolic compounds are common byproducts of many industrial manufacturing processes and are considered as major contaminants, with high toxicity in humans and the environment [1]. Certain phenolic compounds, known as neurotransmitters, also play important roles in humans; among them, dopamine and epinephrine have been intensively studied because of their vital roles in the central nervous system, as well as renal, cardiovascular, and hormonal systems. Abnormally high or low levels of these neurotransmitters are indicative of serious illness and disease, including myocardial infarction, schizophrenia, Parkinson’s disease, and Alzheimer’s disease [2–5]. Therefore, their selective, quantitative, and sensitive determination is required for evaluating health risks to effectively prevent or treat serious physiological symptoms, as well as to understand their biological functions and mechanisms.

To address this need, several instrument-based analytical methods have been utilized, including high-performance liquid chromatography (HPLC), mass spectrometry (MS), and HPLC-MS [6, 7]. Several micro/nanoparticles-assisted analytical strategies have also been reported [8–10]. Although these strategies enable the sensitive determination of target phenolic compounds, they generally require relatively tedious pre- and post-treatment, as well as long operating times, skilled technicians, and expensive instrumentation, all of which make the assay difficult, particularly for use in point-of-care testing (POCT) environments [11, 12]. For more convenient identification, colorimetric, fluorescent, and electrochemical biosensing methods that involve specific probes, such as colorimetric or fluorescent dyes, electron mediators, nanoparticles including quantum dots, gold nanoparticles, or redox-active cerium oxide nanoparticles, and enzymes, such as peroxidase, tyrosinase, or laccase, have been extensively studied [13, 14]. Among them, enzyme-based strategies have attracted wide interest because they can offer simple, reliable, and sensitive detection by enzymatic catalysis to determine target phenols selectively and quantitatively. However, the inevitable limitations of enzymes, such as intrinsic instability during long-term operation and storage, as well as high production/purification costs, have significantly hampered their practical utilization [15]. Correspondingly, there is a significant incentive to develop an innovative strategy to overcome the limitations of natural enzymes, and thus, develop more efficient methods for the convenient, reliable, and sensitive detection of these compounds.

In this regard, nanomaterial-based enzyme mimics (nanozymes) with catalytic activity toward phenolic compounds have been reported. Among these, laccase-mimicking nanozymes have attracted recent attention owing to their ability to selectively oxidize phenols with greatly improved stability [16, 17]. Laccase-like nanozymes developed to date have generally contained copper elements due to their contribution to laccase-like catalytic action, such that natural laccase has four copper elements in its active site. For example, copper-based chemical complexes conjugated with imidazole [18] or porphyrin [19] were reported. However, their activities were considerably lower than that of natural one. Nanozymes containing copper elements conjugated with specific biomolecules, able to efficiently mimic the biomolecule-based active site of natural laccase, were also demonstrated to yield high catalytic activity with improved stability, such as guanosine monophosphate-coordinated copper-containing metal–organic frameworks [20], cysteine–histidine–copper hybrid nanoparticles [21], and guanine-rich single-stranded DNA–copper hybrid nanoflowers [22]. Although these examples demonstrate the potential of laccase-mimicking nanozymes, further research is required to develop more efficient laccase-mimicking nanozymes, which will facilitate real applications, particularly in POCT environments.

Herein, we have developed a new laccase-mimicking nanozyme, prepared by incubating amine-functionalized manganese dioxide nanoflowers (MnO_2_ NFs), which were rapidly synthesized by reducing potassium permanganate, with copper sulfate in phosphate buffered saline (PBS) for three days at room temperature (RT), to yield hierarchical manganese dioxide–copper phosphate hybrid nanoflowers (H–Mn–Cu NFs). Based on the synergistic catalytic action between Mn and Cu and the large surface area, the nanoflowers were demonstrated to have efficient laccase-like activity, yielding a considerably higher *V*_*max*_ (~ 400%) and a lower *K*_*m*_ (~ 85%) than those of natural laccase, with significantly high operational stability. Based on the advantageous characteristics of the developed nanoflowers, paper microfluidic devices were fabricated, enabling convenient colorimetric determination of phenolic neurotransmitters, including dopamine and epinephrine. Using nanoflower-incorporated paper devices, tiny amounts of clinically important phenolic neurotransmitters were conveniently and effectively determined by simply dropping samples onto the devices.

## Materials and methods

### Materials

Potassium permanganate (KMnO_4_, ≥ 99%), citric acid (≥ 99.5%), hydrochloric acid (HCl, 37%), copper(II) sulfate pentahydrate (≥ 98%), laccase from *Trametes versicolor* (≥ 0.5 U/mg), bovine serum albumin (BSA, ≥ 96%), horseradish peroxidase (HRP, ≥ 250 U/mg), phosphate buffered saline (PBS), 2-(N-morpholino)ethanesulfonic acid (MES, ≥ 99%), 3-aminopropyl triethoxysilane (APTES, 99%), 2,4-dichlorophenol (2,4-DP, ≥ 98%), 4-aminoantipyrine (4-AP, ≥ 97%), 2,2′-azino-bis(3-ethylbenzothiazoline-6-sulfonic acid) diammonium salt (ABTS, ≥ 98%), hydrogen peroxide (H_2_O_2_, 30% aqueous solution), dopamine hydrochloride (≥ 98%), epinephrine (≥ 99%), phenol (99.0-100.5%), bisphenol A (≥ 99%), hydroquinone (≥ 99%), catechol (≥ 95%), 2-naphthol (≥ 99%), crystal violet (≥ 96%), neutral red (≥ 90%), and rhodamine B (≥ 95%) were purchased from Sigma-Aldrich (St. Louis, MO, USA). Deionized water purified using a Milli-Q Purification System (Millipore, Darmstadt, Germany) was used to prepare all solutions. All chemicals were of analytical grade or higher and used as received without further purification.

### Synthesis and characterization of MnO_2_ NFs, amine-functionalized MnO_2_ NFs, and H–Mn–Cu NFs

MnO_2_ NFs were synthesized according to a previous study, with some modifications [23]. Briefly, KMnO_4_ (80 mg) was dissolved in an aqueous HCl solution (1 M, 40 mL). Then, an aqueous citric acid solution (100 mM, 1 mL) was added to the solution and stirred for 30 min at room temperature (RT, 22 °C), yielding a color change from red violet to brown. The resulting MnO_2_ NFs were collected via centrifugation at 10,000 rpm for 5 min, washed with distilled water, and dried at 50 °C under vacuum. Amine-functionalized MnO_2_ NFs were synthesized by dispersing MnO_2_ NFs (150 mg) into a mixture of distilled water (2 mL) and absolute ethanol (300 mL), followed by sonication for 10 min. APTES (0.6 mL) was added to the mixture under constant stirring for 7 h. The resulting amine-functionalized MnO_2_ NFs were collected by centrifugation at 10,000 rpm for 5 min, washed with ethanol, and dried at 50 °C under vacuum for 1 d. H–Mn–Cu NFs were synthesized according to a previously reported self-assembly method, with marginal modifications [24]. Typically, 60 µL of aqueous CuSO_4_ solution (120 mM) was added to 9 mL of PBS (10 mM, pH 7.4) containing the amine-functionalized MnO_2_ NFs (0.1 mg mL^− 1^), followed by three days of incubation at RT. The resulting H–Mn–Cu NFs were then collected using centrifugation at 10,000 rpm for 5 min, washed three times with deionized water, and dried at 50 °C under vacuum. As a control, Cu_3_(PO_4_)_2_ precipitates were prepared by incubating a CuSO_4_ solution in PBS for three days at RT, as previously reported [25].

The size, morphology, and elemental composition of the synthesized nanoflowers were analyzed using scanning electron microscopy (SEM) (Magellan 400 microscope; FEI Co., Cambridge, UK) with an energy-dispersive X-ray spectrometer (EDS; Bruker, Billerica, MA). For SEM, a suspension of nanoflowers was dropped on a silicon wafer and dried overnight at RT. Fourier transform infrared (FT-IR) spectra and X-ray diffraction (XRD) patterns of the MnO_2_ NFs, amine-functionalized MnO_2_ NFs, H–Mn–Cu NFs, and Cu_3_(PO_4_)_2_ precipitates prepared by incubating only copper sulfate in PBS without MnO_2_ NFs were obtained using an FT-IR spectrophotometer (FT/IR-4600; JASCO, Easton, MD) and an X-ray diffractometer (D/MAX-2500; Rigaku Corporation, Tokyo, Japan), respectively. The specific surface area, pore diameter distribution, and pore volume were obtained from N_2_ physisorption isotherms obtained with a physisorption analyzer (3Flex; Micromeritics, GA, USA) using the Brunauer–Emmett–Teller (BET) and Barrett–Joyner–Halenda (BJH) methods. X-ray photoelectron spectroscopy (XPS) (Sigma Probe, Thermo Scientific, WI, USA) was performed to investigate the electronic states of the Mn and Cu within the H-Mn-Cu NFs. The elemental ratio between Mn and Cu within the H-Mn-Cu NFs was determined via an inductively coupled plasma mass spectrometry (ICP-MS; Agilent 7700 S, CA, USA) analysis.

### Determination of laccase-mimicking activity of H–Mn–Cu NFs

Laccase-like activity was measured using the chromogenic reaction of phenolic compounds with 4-AP as follows: First, 2,4-DP (1 mg mL^1^, 100 µL) was mixed with 4-AP (1 mg mL^− 1^, 100 µL) in MES buffer (50 mM, pH 6.8, 700 µL). Free laccase or H–Mn–Cu NFs (1 mg mL^− 1^, 100 µL) were then added. After reacting for 40 min at RT, the mixture was centrifuged at 10,000 rpm for 2 min, and the absorbance of the supernatant was recorded in scanning mode or at 510 nm using a microplate reader (Synergy H1; BioTek, VT, USA, at the Core-facility for Bionano Materials in Gachon University). Other phenolic substrates (phenol, bisphenol A, hydroquinone, catechol, 2-naphthol, and dopamine) were used as target compounds instead of 2,4-DP; the other assay procedures were the same as those described above.

The effects of pH on the laccase-like activity of H–Mn–Cu NFs were examined following the same procedures but using MES buffer solutions prepared from pH 3 to 10. The effects of incubation temperature on the activity of H–Mn–Cu NFs were also explored following the same procedures but incubated under diverse temperature conditions (4–80 °C). The relative activity (%) was calculated using the ratio of measured activity to the standard activity, measured at pH 6.8 and RT. Stabilities for pH, temperature, and ionic strength of H–Mn–Cu NFs and free laccase were evaluated by incubating them in aqueous buffer (MES, 50 mM) at different pH values (pH 3–10) for 5 h, different temperatures (4–80 °C) for 3 h, and different NaCl concentrations (0, 62.5, 125, 250, and 500 mM) for 10 h, followed by measurement of the residual activities using standard assay methods. The long-term operational stabilities of H–Mn–Cu NFs and free laccase were measured by assessing their daily activities during their incubation at RT under mild shaking conditions. The relative activity (%) was calculated as the ratio of residual activity to the initial activity of each sample.

Steady-state kinetic parameters were evaluated by performing the laccase-mediated reaction at RT in a 1.5-mL tube with H–Mn–Cu NFs or free laccase (both at concentrations of 0.1 mg mL^− 1^) in MES buffer (50 mM, pH 6.8). Epinephrine at various concentrations (9.4, 18.7, 37.5, 75, 150, 300, and 600 µM) was added to 1 mL of reaction buffer. After the substrates were mixed, the color changes were monitored in kinetic mode at 485 nm. The kinetic parameters were calculated based on the Michaelis–Menten equation: *ν* = *V*_*max*_ × [S] / (*K*_*m*_ + [S]), where *ν* is the initial velocity, *V*_*max*_ is the maximal velocity, [S] is the concentration of the substrate, and *K*_*m*_ is the Michaelis constant.

To evaluate the dopamine detection sensitivity of the H–Mn–Cu NFs, dopamine at various concentrations (100 µL) was mixed with 4-AP (1 mg mL^− 1^, 100 µL) and H–Mn–Cu NFs (1 mg mL^− 1^, 100 µL) in MES buffer (50 mM, pH 6.8, 700 µL), followed by incubation for 40 min at RT. After the reaction, the mixture was centrifuged at 10,000 rpm for 2 min, and the absorbance of the supernatant was recorded at 510 nm. To measure the detection sensitivity for epinephrine, epinephrine at diverse concentrations (100 µL) was mixed with H–Mn–Cu NFs (1 mg mL^− 1^, 100 µL) in MES buffer (50 mM, pH 6.8, 800 µL). The other procedures were the same as those described for the detection of dopamine, except that the absorbance at 485 nm, which corresponds to the oxidized epinephrine, was measured rather than 510 nm. The limit of detection (LOD) values were calculated according to the equation LOD = 3 S / K, where *S* is the standard deviation of the blank absorbance signals, and *K* is the slope of the calibration plot.

### Degradation of dyes by H–Mn–Cu NFs or free laccase

The dye degradation efficiencies of H–Mn–Cu NFs and free laccase were assessed using crystal violet (CV), neutral red (NR), and rhodamine B (RB) as model dyes. First, H–Mn–Cu NFs or laccase (1 mg mL^− 1^, 1 mL) was mixed with the dye solution [9 mL at concentrations of 2.5 mg mL^− 1^ (CV), 7.5 mg mL^− 1^ (NR), or 1.5 mg mL^− 1^ (RB)]. The mixture was incubated in the dark with gentle shaking at RT. The dye degradation efficiencies of CV, NR, and RB were analyzed by measuring the absorption intensities at 590, 523, and 543 nm, respectively, at predetermined time points, using a microplate reader. Matrix-assisted laser desorption/ionization – time of flight (MALDI-TOF, Bruker autoflex maX, Bruker Daltonics, MA, USA) mass spectrometry was performed to confirm the degradation of CV, NR, and RB by the incubation with H-Mn-Cu NFs.

### H–Mn–Cu NFs-embedded paper microfluidic devices for colorimetric determination of phenolic neurotransmitters

Paper microfluidic devices, including H–Mn–Cu NFs, were constructed using a wax printing method [26]. The pattern was first designed using AutoCAD 2018, followed by printing wax on Whatman chromatography paper (grade 1) with a wax printer (ColorQube 8570DN; Xerox, Japan). The printed paper was placed on a hot plate at 170ºC for 2 min to melt the wax and then cooled at RT to form hydrophobic barriers.

For the detection of both dopamine and epinephrine on a single device, the microfluidic device was divided into two parts for the detection of dopamine (D) and epinephrine (E). In each part, there were three circular detection zones (6 mm in diameter), microfluidic channels (3 mm in width, and 6 mm in length), one half-circular sample zone (7.5 mm in diameter) for sample injection, and one circular control zone (6 mm in diameter). On the dopamine-detecting part, both H–Mn–Cu NFs and 4-AP were immobilized in the control zone, whereas only H–Mn–Cu NFs were immobilized in the control zone on the epinephrine-detecting part. To detect single neurotransmitters of either dopamine or epinephrine, the device was not divided and consisted of six circular detection zones and one circular sample zone connected to the microfluidic channels. For the dopamine-detecting device, two circular control zones (6 mm in diameter) were prepared, where the first contained both H–Mn–Cu NFs and 4-AP, and the other contained only 4-AP without the nanoflowers. For the epinephrine-detecting device, a circular control zone (6 mm in diameter) was prepared, where only the H–Mn–Cu NFs were immobilized.

To construct paper microfluid devices with incorporated H–Mn–Cu NFs, H–Mn–Cu NFs (10 mg mL^− 1^, 2 µL) were dropped onto the detection and control zones of the devices. 4-AP (5 mg mL^− 1^, 2 µL) was consecutively dropped on the dopamine detection zones and control zones. The paper device was then dried at 50 °C for 5 min. To detect phenolic neurotransmitters, 20 µL of the sample solution containing dopamine or epinephrine was dropped twice onto both parts of the half-circular sample zone or 40 µL of sample solution was dropped once onto the circular sample zone to detect single phenolic neurotransmitters of either dopamine or epinephrine. After 10 min, the resulting devices were directly used to obtain images with a smartphone (Galaxy S8 NOTE; Samsung, Korea), followed by conversion to a yellow scale, which was subjected to quantitative image processing using the ImageJ software (NIH).

## Results and discussion

### Construction of laccase-mimicking H–Mn–Cu NFs

**Fig. 1:**
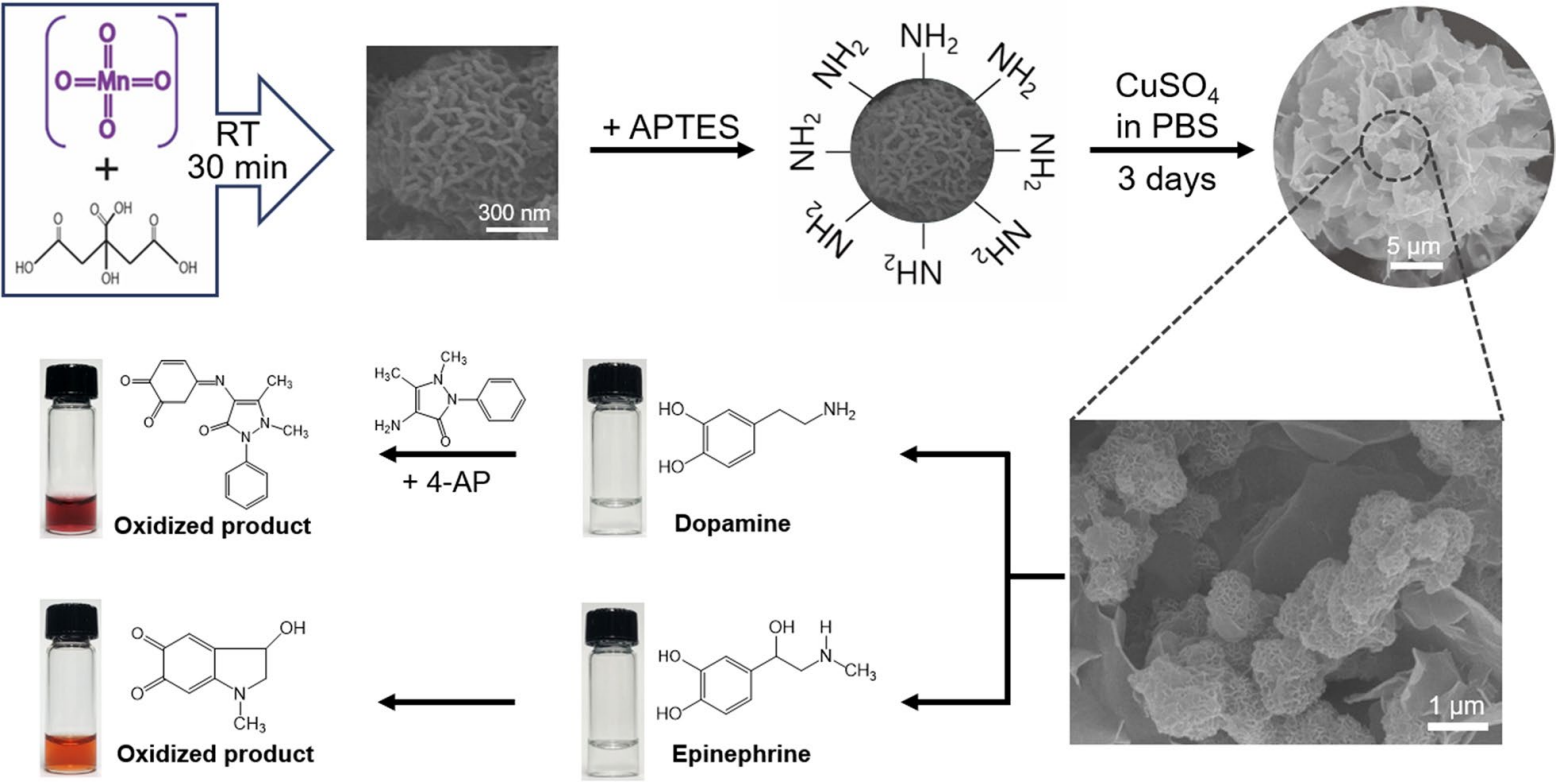
Schematic illustration of the preparation of laccase-mimicking H–Mn–Cu NFs and their application to colorimetrically detect dopamine and epinephrine

The overall strategy for the synthesis of laccase-mimicking H–Mn–Cu NFs and their application to colorimetric detection of phenolic neurotransmitters, including dopamine and epinephrine, is illustrated in Fig. 1. MnO_2_ NFs were first prepared by rapidly reducing KMnO_4_ using citric acid, followed by amine functionalization of their surfaces using APTES. Copper(II) sulfate solution in PBS was further added and incubated for three days at RT to induce coordination interactions between the amine moieties of MnO_2_ NFs and copper ions, nucleation of copper phosphate crystals, and their anisotropic self-assembly to form a nanostructured flower-like morphology, consequently yielding H–Mn–Cu NFs. Since Cu elements are known to be essential for mimicking laccase activity, as well as Mn elements, enabling efficient laccase-like phenol oxidation [7, 21], we hypothesized that the H–Mn–Cu NFs, where both Mn and Cu elements coexist throughout the nanoflowers with a large surface area, thereby increasing the number of catalytic events, would be an efficient laccase-mimicking nanozyme. Specifically, in the presence of dopamine in the sample solution, the nanoflowers catalyze its oxidation to further react with 4-AP to produce a violet-colored adduct. Epinephrine in the sample solution is directly oxidized by the laccase-like activity of the nanoflowers, producing an orange-colored product. We envisioned that the H–Mn–Cu NF-mediated colorimetric detection strategy for dopamine and epinephrine could be extended to construct paper microfluidic devices that can conveniently quantify the target phenolic neurotransmitters in POCT environments only with the help of an image acquired using a conventional smartphone.

**Fig. 2:**
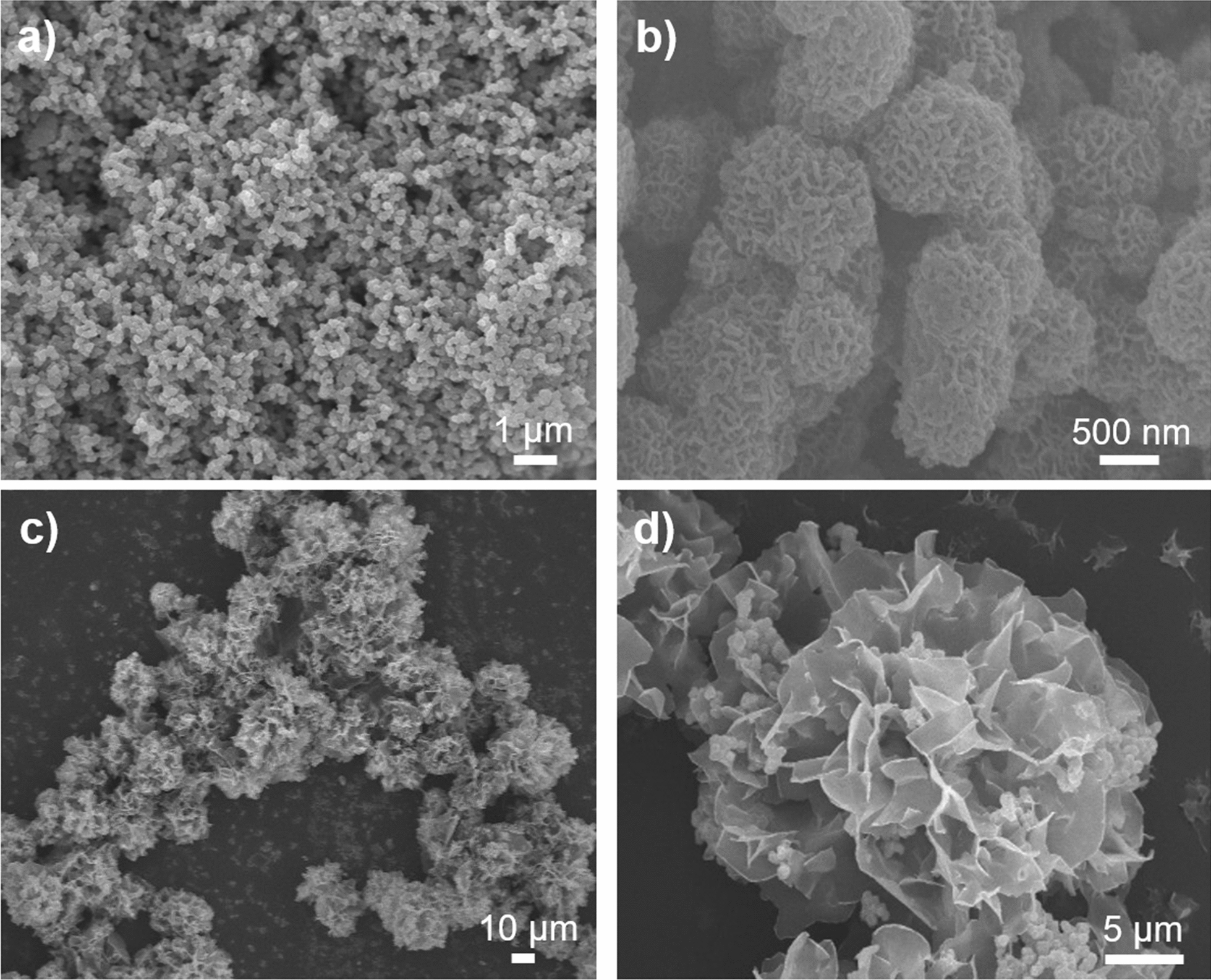
SEM images of **a**, **b** MnO_2_ NFs and **c**, **d** H–Mn–Cu NFs

The morphologies of the initial MnO_2_ NFs and H–Mn–Cu NFs after three days of incubation were investigated (Fig. 2). The SEM images of both MnO_2_ NFs and H–Mn–Cu NFs showed flower-like spherical structures but their sizes were ~ 0.5 and ~ 30 μm, respectively, possibly due to the anisotropic three-day growth from MnO_2_ NFs to H–Mn–Cu NFs. Furthermore, in the enlarged SEM image shown in Fig. 1, many MnO_2_ NFs are located on a petal of H–Mn–Cu NFs, proving the efficient incorporation of MnO_2_ NFs within copper phosphate-based nanoflowers. The time-dependent morphology of the H–Mn–Cu NFs was monitored by obtaining their SEM images during growth (see Additional file 1: Fig. S1). At the initial stage, only MnO_2_ NFs were observed. However, at 12 h of incubation after adding copper sulfate solution, larger particles with sharp petal-like structures could be observed. As the incubation time increased, size increased as well as flower-like shape evolved, and at three days of incubation, the blooming process was completed to yield final H–Mn–Cu NFs having a large surface-to-volume ratio. The progressive growth process is very similar to that of conventional organic–inorganic hybrid nanoflowers [24], which involves mineralization between the nitrogen elements of amide/amine moieties in biomolecules and metal ions and subsequent anisotropic growth by the precipitation of metal phosphate crystals, indicating that the amine-functionalized MnO_2_ NFs could efficiently replace the biomolecules in organic–inorganic hybrid nanoflowers to bloom H–Mn–Cu NFs.

To gain insights into the structure of the H–Mn–Cu NFs, they were subjected to SEM with EDS, XRD, FT-IR, and BET analysis. Elemental mapping showed the presence of representative elements, corresponding to copper phosphate (Cu, P, and O) and MnO_2_ (Mn and O), which were clearly observed throughout the nanoflowers (Additional file 1: Fig. S2). The peaks in the XRD patterns of the H–Mn–Cu NFs matched those of the standard crystal structures of γ-MnO_2_ (JCPDS no. 14–0644) and Cu_3_(PO_4_)_2_·3H_2_O (JCPDS no. 00-022-0548) [27], proving that both crystalline MnO_2_ and Cu_3_(PO_4_)_2_ were present within the NFs (Additional file 1: Fig. S3a). The chemical structures of the H–Mn–Cu NFs were compared with those of the MnO_2_ NFs, amine-functionalized MnO_2_ NFs, and Cu_3_(PO_4_)_2_ precipitates using FT-IR (Additional file 1: Fig. S3b). The peaks observed at 960–1200 cm^− 1^ in the spectra of H–Mn–Cu NFs were attributed to P–O and P =O vibrations, clearly showing the existence of phosphate groups in the nanoflowers, which were observed with Cu_3_(PO_4_)_2_ precipitates but not with MnO_2_ NFs and amine-functionalized MnO_2_ NFs owing to the absence of phosphate groups. According to the BET analyses of MnO_2_ NFs, amine-functionalized MnO_2_ NFs, and H–Mn–Cu NFs, the surface area and pore volume were determined to be 62.0 m^2^ g^− 1^ with 0.026 cm^3^ g^− 1^, 14.8 m^2^ g^− 1^ with 0.006 cm^3^ g^− 1^, and 163.1 m^2^ g^− 1^ with 0.066 cm^3^ g^− 1^, respectively, confirming the highly porous structure with large surface-to-volume ratio of the prepared H–Mn–Cu NFs (Additional file 1: Fig. S3c).

XPS characterizations were conducted to analyze the oxidation states of Cu and Mn elements within the H-Mn-Cu NFs. The full scan spectrum reveals the appearance of Cu, Mn, O, N, C, and P, which are consistent with the elements of Cu_3_(PO_4_)_2_ and amine-functionalized MnO_2_ NFs within the NFs (Additional file 1: Fig. S4). In the high-resolution XPS spectra of Cu ions, the peaks at 933.5 and 953.4 eV represented the Cu 2p_3/2_ and Cu 2p_1/2_ electrons of Cu^+^, respectively (Additional file 1: Fig. S5a, b). The higher binding peaks at 938.4 and 957.9 eV indicated the presence of Cu^2+^. As demonstrated previously [21, 22], the existence of both Cu^+^ and Cu^2+^ is of great importance to the level of catalytic activity of laccase mimics. Importantly, we found that the Cu^+^/Cu^2+^ ratio of H-Mn-Cu NFs (~ 350%) was much higher than that of Cu_3_(PO_4_)_2_ precipitates (~ 70%), presumably due to the affirmative interaction between the amine-functionalized MnO_2_ NFs and copper ions, resulting in the reduction of Cu^2+^ into Cu^+^. Moreover, the high-resolution spectra of Mn ions showed the presence of both Mn^3+^ and Mn^4+^, as demonstrated by the binding peaks at 641.0 and 652.4 eV, corresponding to the Mn 2p_3/2_ and Mn 2p_1/2_ electrons of Mn^3+^, respectively, while the occurrence of Mn^4+^ was attainable from the peaks at 642.6 and 654.1 eV (Additional file 1: Fig. S5c, d) [28]. Interestingly, the Mn^3+^/Mn^4+^ ratio of H-Mn-Cu NFs (~ 190%) was considerably higher than that of bare MnO_2_ NFs (~ 80%). Since the reduced forms of Mn could accelerate the oxidation, nanozymes with higher Mn^3+^/Mn^4+^ values could exhibit higher laccase-like activity [29]. Overall, the higher amount of reduced Cu and Mn species within the H-Mn-Cu NFs might be a theoretical basis for their higher laccase-like activity.

### Highly efficient laccase-mimicking activity of H–Mn–Cu NFs

**Fig. 3:**
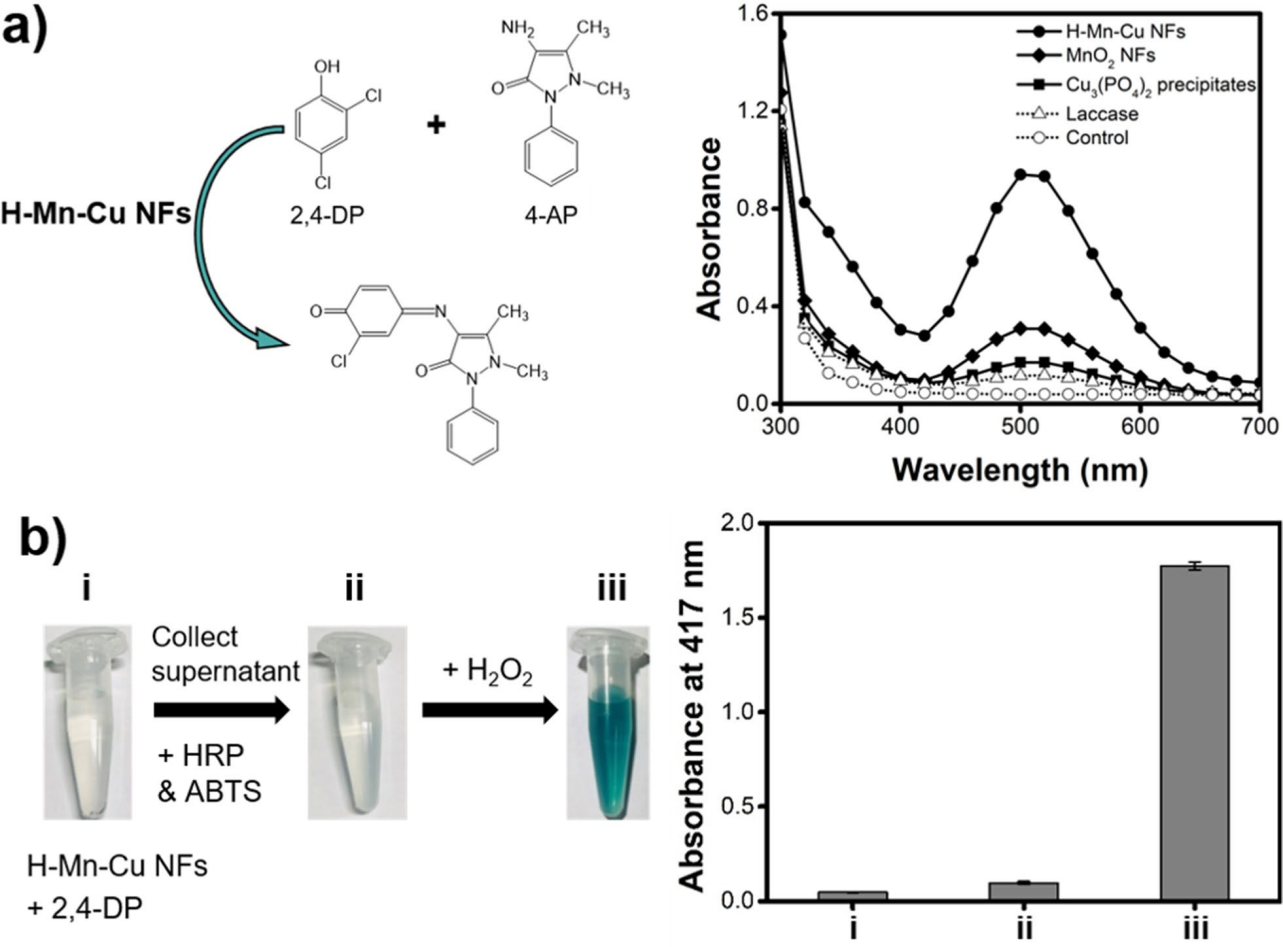
**a** Catalytic reaction of 2,4-DP and 4-AP to produce colored adduct by laccase-mimicking H–Mn–Cu NFs with the absorption spectra for comparing the laccase-like activity of H–Mn–Cu NFs with those of control samples such as MnO_2_ NFs, Cu_3_(PO_4_)_2_ precipitates, and free laccase. **b** Photographs and the corresponding absorption intensities for confirming the laccase-like activity of H–Mn–Cu NFs rather than other oxidase activity. [Sample specifications: (i) H–Mn–Cu NFs reacted with 2,4-DP, (ii) supernatant with ABTS and HRP, added after the centrifugation of (i), and (iii) after adding H_2_O_2_ into (ii)]

The laccase-like activities of the H–Mn–Cu NFs were examined by performing a chromogenic reaction of 2,4-DP and 4-AP, and the responses were monitored using absorption spectroscopy (Fig. 3a). With the catalytic action of laccase, 2,4-DP was oxidized and further reacted with 4-AP, yielding a reddish-purple adduct with an absorption peak at approximately 510 nm. The experimental results clearly showed that the H–Mn–Cu NFs had a vivid laccase-like activity and yielded the highest colorimetric responses among the other control samples, such as MnO_2_ NFs, Cu_3_(PO_4_)_2_ precipitates, and free laccase. This revealed the synergistic effect between Cu and Mn elements to mimic the laccase-like catalytic action as well as the affirmative effect of a large surface-to-volume ratio, which increased the number of catalytic events. Elemental ratio between Mn and Cu within the H-Mn-Cu NFs was determined to be approximately 1:1 via ICP-MS analysis, indicating that both elements could equally contribute to the synergistic enhancement in the catalytic activity. H–Mn–Cu NFs were further confirmed to have laccase activity to convert oxygen into water, while other oxidases produce H_2_O_2_ (Fig. 3b) [20]. For this, H–Mn–Cu NFs were incubated with 2,4-DP for 40 min, and the supernatant was collected by centrifugation-mediated separating the H–Mn–Cu NFs. Following this, the peroxidase substrates ABTS and HRP were added to the supernatant; however, color change was not observed. After adding H_2_O_2_, green color corresponding to the oxidized ABTS was observed, clearly indicating that the H–Mn–Cu NFs are laccase mimics and not other oxidases.

Observations of the effects of pH and temperature on the laccase-like activity of H–Mn–Cu NFs revealed that neutral-basic pH ranging from 6 to 10 and a wide range of temperatures from 4 to 80 °C were acceptable assay conditions, yielding over 80% of the activity under standard conditions (Additional file 1: Fig. S6). For practical convenience, pH 6.8 and RT were adopted for further studies. H–Mn–Cu NFs are stable over a wide range of pH values (pH 3‒10), temperatures (4‒80 °C), ionic strengths (up to 500 mM NaCl), and 10 days of operating conditions (Additional file 1: Fig. S7). In contrast, free laccase was not stable under extreme conditions and longer incubation time. The increased activity of H–Mn–Cu NFs at high ionic strengths was presumed to result in more efficient binding of the substrate to the nanoflowers, whereas free laccase yielded a significant loss of its activity owing to the inhibition by chloride ions from their binding to the nuclear copper sites [21]. The noticeably high stability of H–Mn–Cu NFs is beneficial for their use as a feasible alternative to natural enzymes for versatile applications.

To clearly demonstrate the laccase-like activity of the H–Mn–Cu NFs, steady-state kinetic parameters were determined using epinephrine as a single phenolic substrate and compared with those of free laccase and recently reported laccase mimics (Table 1 and Additional file 1: Fig. S8). The apparent *K*_*m*_ value of H–Mn–Cu NFs was noticeably lower than that of free laccase and most of the previous laccase mimics, indicating that the mass transfer limitation of H–Mn–Cu NFs is not serious and is even lower than that of free laccase. Moreover, the *V*_*max*_ value of H–Mn–Cu NFs was 4-fold higher, indicating that H–Mn–Cu NFs are more active laccase-like catalysts than natural laccases.

**Table 1:** Comparison of kinetic parameters of H–Mn–Cu NFs with those of free laccase and previous reports, using epinephrine as a substrate

Catalyst	Concentration (mg mL^− 1^)	*K*_*m*_ (mM)	*V*_*max*_ (mM min^− 1^)	References
H–Mn–Cu NFs	0.1	0.14 ± 0.02	1.3 ± 0.12	This work
Free laccase	0.1	0.17 ± 0.03	0.3 ± 0.04	
Cu/H_3_BTC MOF^a^	0.1	0.07	9.4 × 10^− 2^	[30]
CH–Cu^b^	0.1	0.58	2.7 × 10^− 2^	[21]

^a^ Metal–organic frameworks prepared from copper and 1,3,5-benzene tricarboxylic acid.

^b^ Cysteine–histidine–copper hybrid nanoparticles.

**Fig. 4:**
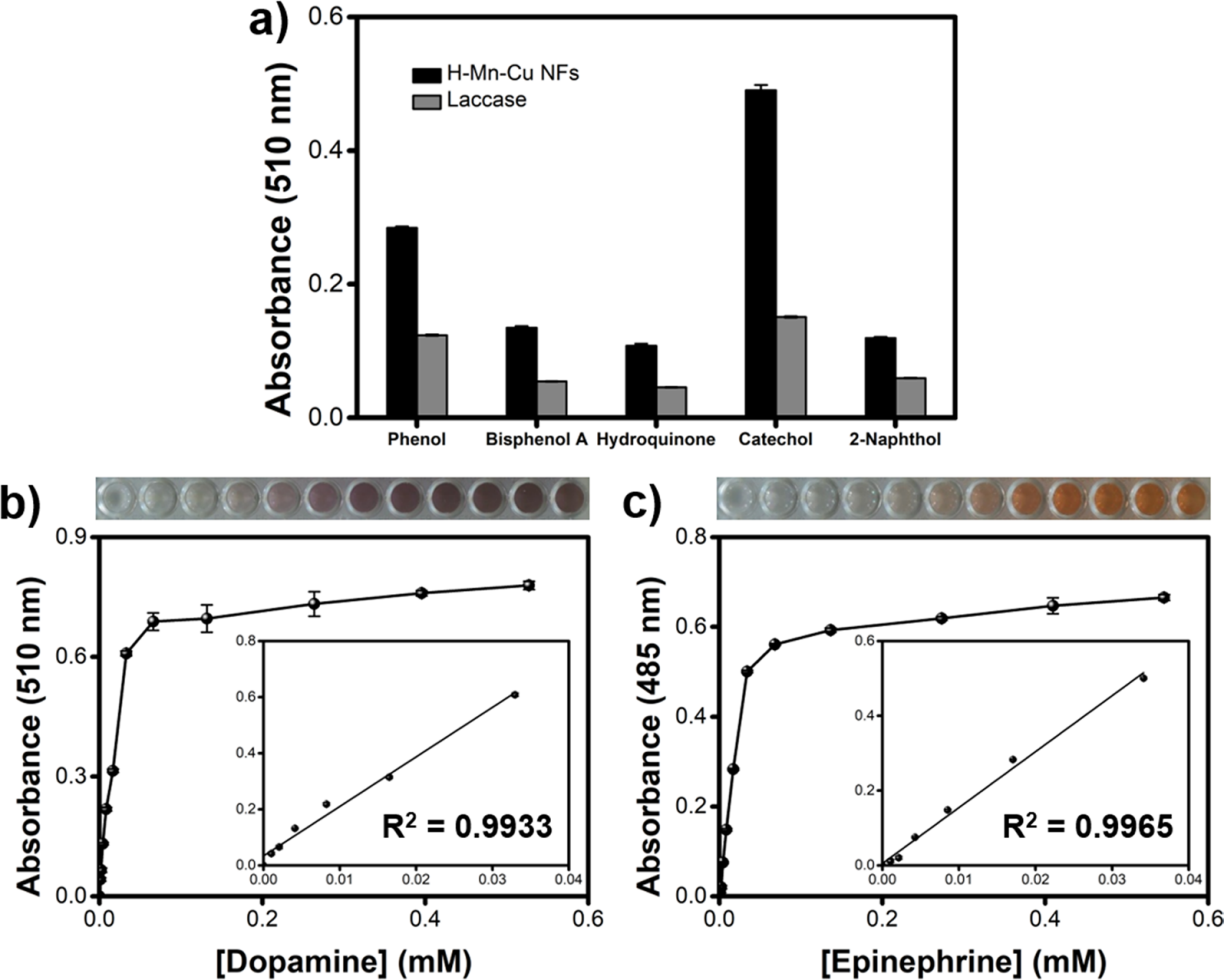
**a** Comparison of catalytic activities of H–Mn–Cu NFs and free laccase to oxidize five different phenolic substrates (phenol, bisphenol A, hydroquinone, catechol, and 2-naphthol). Dose–response curves and linear calibration plots for determining **b** dopamine and **c** epinephrine by well plate-based assay using H–Mn–Cu NFs. Photographs show the real images of the assay with target concentrations of 0–0.55 mM. Error bars represent standard deviations obtained from four independent measurements

We then investigated the substrate diversity of H–Mn–Cu NFs to catalyze the oxidation of diverse phenolic substrates. The results showed that H–Mn–Cu NFs catalyzed five employed phenols, namely phenol, bisphenol A, hydroquinone, catechol, and 2-naphthol, with higher catalytic activity than that of free laccase, over 2-fold in all cases (Fig. 4a). Inspired by the large substrate diversity, H–Mn–Cu NFs were applied to quantitatively determine two important phenolic neurotransmitters, dopamine and epinephrine, which are closely related to many physiological functions and symptoms. Thus, these are frequently used as reliable markers to diagnose and treat many human diseases, such as allergies, asthma, cardiac arrhythmias, and infarction [31]. We first demonstrated that the H-Mn-Cu NFs exhibited the highest laccase-like activity to detect dopamine among free laccase and the other laccase mimics (Additional file 1: Fig. S9). Through the well plate-based assay with H–Mn–Cu NFs, dopamine and epinephrine were determined quantitatively and sensitively, yielding LODs as low as 84.9 and 100.2 nM, respectively (Fig. 4b and c), using the calibration plots prepared from dose–response curves. With free laccase, dopamine and epinephrine were also linearly determined but their LODs were considerably higher, calculated to be 5.6 and 2.4 µM, respectively (Additional file 1: Fig. S10). These findings demonstrate that H–Mn–Cu NFs can use diverse phenols as their substrates, including dopamine and epinephrine, with higher sensitivity than free laccase.

**Fig. 5:**
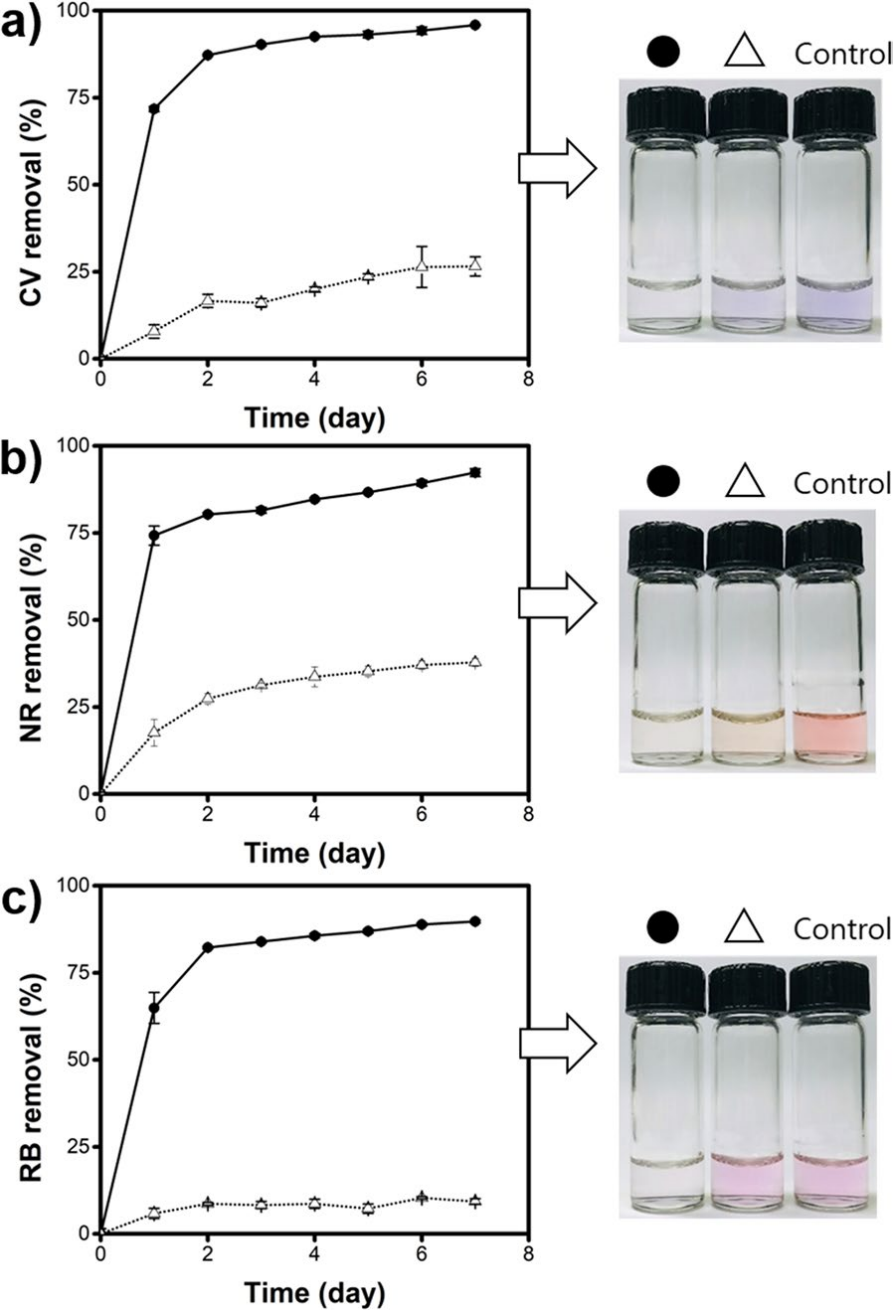
Photographs and the corresponding degradation efficiencies of **a** crystal violet (CV), **b** neutral red (NR), and **c** rhodamine B (RB) by H–Mn–Cu NFs (●) and free laccase (△)

Laccase has also been widely employed for phenolic dye degradation [32], where H–Mn–Cu NFs can be used. To evaluate this applicability, the dye degradation efficiencies of the H–Mn–Cu NFs were assessed using CV, NR, and RB as models and compared with those of free laccase. After a two-day incubation period, over 80% of the dyes lost their original color by simple incubation with H–Mn–Cu NFs at RT, whereas free laccase displayed less than 30% degradation (Fig. 5). H-Mn-Cu NFs-mediated degradation of employed CV, NR, and RB into fragmented products was further confirmed by MALDI-TOF mass spectrometry (Additional file 1: Fig. S11) [33, 34]. Even when the incubation was extended up to 1 week, free laccase yielded less than 40% degradation efficiency, whereas H-Mn-Cu NFs yielded over 90% efficiency, showing their high applicability in dye degradation. The ability of the H–Mn–Cu NFs to efficiently degrade phenolic dyes is highly beneficial for making them effective for environmental applications.

### H–Mn–Cu NFs-embedded paper microfluidic device for the convenient quantification of phenolic neurotransmitters

**Fig. 6:**
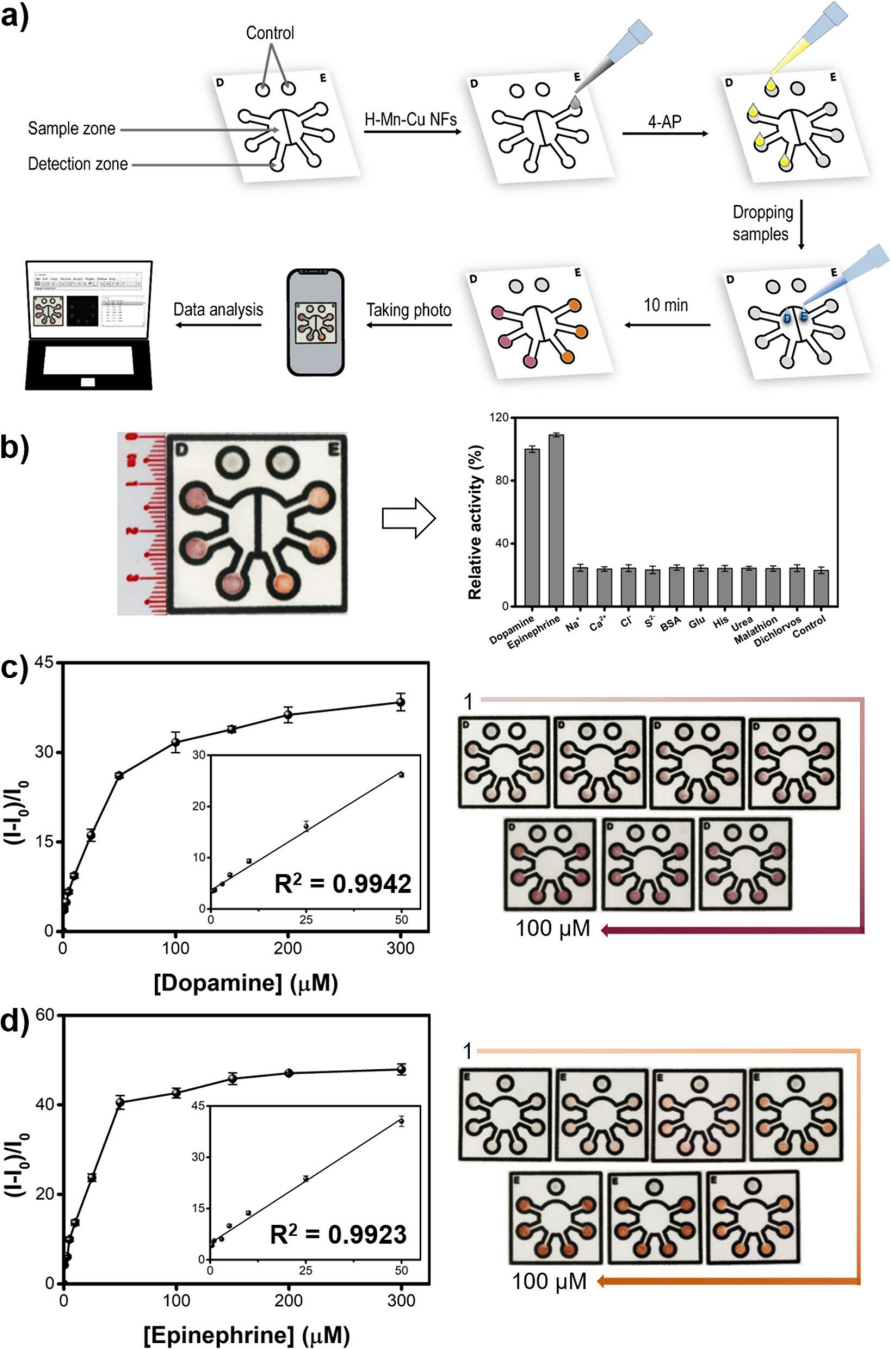
**a** Schematic representation of the construction and utilization of H–Mn–Cu-NFs-incorporated paper microfluidic device for the visual determination of phenolic neurotransmitters. **b** Selectivity of H–Mn–Cu NFs-incorporated paper microfluidic device for simultaneous detection of dopamine (D) and epinephrine (E). Both dopamine and epinephrine were used at a concentration of 20 µM, whereas all control samples were used at a concentration of 200 µM. Sensitivity of H–Mn–Cu NFs-incorporated paper microfluidic device for detecting **c** dopamine and **d** epinephrine. Dose–response curves, the corresponding linear calibration plots, as well as the real photographs of the devices were presented for detecting each target phenolic neurotransmitter at seven representative concentrations (1, 3, 5, 10, 25, 50, and 100 µM)

Based on the high laccase-like activity and stability of the H–Mn–Cu NFs, a new and efficient paper microfluidic device was developed that enabled convenient colorimetric quantification of phenolic neurotransmitters, including dopamine and epinephrine. We designed and constructed a simple wax-printed paper device that was divided into two parts for the simultaneous detection of both dopamine and epinephrine (Fig. 6a). Each part consisted of one half-circular sample zone and three detection zones. To detect dopamine (D side), both 4-AP and H–Mn–Cu NFs were impregnated within the detection zones, and to detect epinephrine (E side), only H–Mn–Cu NFs were impregnated. By dropping ~ 20 µL of sample solution twice onto each divided sample zone, specific violet and orange-colored signals, corresponding to dopamine and epinephrine, were generated during a 10 min reaction at RT. Importantly, the color signals within the detection zones were quantified by taking images using a smartphone, followed by image processing using ImageJ software. In the case of detecting single phenolic neurotransmitters of either dopamine or epinephrine, whole-paper devices consisting of one circular sample zone with six detection zones were constructed and utilized for higher detection precision.

First, we compared the color signals of the paper devices for detecting dopamine and epinephrine with those prepared using MnO_2_ NFs, Cu_3_(PO_4_)_2_ precipitates, and free laccase (Additional file 1: Fig. S12). The results clearly show that the H–Mn–Cu NF-based paper microfluidic device yielded considerably darker violet and orange colors, corresponding to the target dopamine and epinephrine, respectively, as observed in the solution-based assay experiments. The fabrication and sensing conditions for the square-shaped paper microfluidic device (3.7 cm in side) were optimized, such as wax barrier thickness to prevent the flow leakage and the other parameters (reaction time and employed concentrations of 4-AP and H–Mn–Cu NFs) to maximize the color signals (Additional file 1: Figs. S13, S14, and S15). Under the optimized conditions, target phenolic neurotransmitters, including dopamine and epinephrine, were selectively detected, whereas negligible color intensity was detected from the interfering substances, where metal and nonmetal ions (Na^+^, Ca^2+^, Cl^−^, S^2−^), physiological molecules (BSA, glucose, histidine, and urea), and organophosphorus compounds (malathion and dichlorvos) were used, even at 10-fold higher concentrations (Fig. 6b). With increasing concentrations of dopamine and epinephrine, the respective color intensities gradually increased without any significant variation (Fig. 6c and d). The analysis of dose–response curves determined the LODs for dopamine and epinephrine as 54.0 nM and 34.5 nM, respectively, with the linear range of 0.5–50 µM for the detection of both neurotransmitters, with R^2^ > 0.99 in all cases (Fig. 6c and d). As presented in Table S1, The LOD and linear range values of our H-Mn-Cu NF-based paper microfluidic device were among the best for the colorimetric detection of phenolic neurotransmitters [35–37]. Considering the physiological levels of dopamine and epinephrine in human serum, the developed paper device can also be used to discriminate between patients and normal [13, 14]. Furthermore, the paper device showed excellent stability for up to two months when stored at RT (Additional file 1: Fig. S16), which is beneficial for practical applications.

We also demonstrated the clinical utility of the H–Mn–Cu NF-based paper microfluidic device for simultaneous quantification of dopamine and epinephrine in spiked human serum samples. Three representative levels of dopamine and epinephrine (5, 10, and 20 µM) in 2-fold diluted spiked serum were precisely and accurately determined, yielding coefficients of variation (CV) in the range of 2.7‒4.8% and recoveries of 97.2‒103.9% (Table S2). These results demonstrate that the developed H–Mn–Cu NF-based paper microfluidic device can serve as a promising analytical tool for the convenient determination of phenolic neurotransmitters in instrumentation-free POCT environments.

## Conclusions

In this study, we developed an innovative laccase-mimicking nanozyme, H–Mn–Cu NFs, with considerably higher catalytic activity than natural laccase owing to the synergistic catalysis between Cu and Mn throughout the large nanoflower matrices. The H–Mn–Cu NFs accepted diverse phenolic compounds as their substrate, as well as exhibited excellent stability over the ranges of reaction environments and prolonged incubation time. By applying the H–Mn–Cu NFs to paper microfluidic devices, target phenolic neurotransmitters, including dopamine and epinephrine, were conveniently quantified using real images taken by a smartphone, along with excellent sensitivity, selectivity, stability, and reliability. This study provides a foundation for continued efforts toward the development of new nanozymes, as well as nanozyme-based paper microfluidic devices with significant potential for diverse biosensing applications, particularly in POCT environments.

## Supplementary Information

**Additional file 1: Fig. S1**. SEM images of H–Mn–Cu NFs after incubation for (a) 0, (b) 12, (c) 24, (d) 48, and (e) 72 h. Insets represent high-resolution SEM images. **Fig. S2**. Elemental mapping of H–Mn–Cu NFs. **Fig. S3**. (a) XRD pattern and (b) FT-IR spectra of MnO_2_ NFs, amine-functionalized (APTES)-MnO_2_ NFs, Cu_3_(PO_4_)_2_ precipitates, and H–Mn–Cu NFs. (c) BET surface areas of MnO_2_ NFs, APTES-MnO_2_ NFs, and H–Mn–Cu NFs. **Fig. S4**. XPS full scan spectrum of H-Mn-Cu NFs. **Fig. S5**. High-resolution XPS spectra of Cu for (a) H-Mn-Cu NFs and (b) Cu_3_(PO_4_)_2_ precipitates, and Mn for (c) H-Mn-Cu NFs and (d) bare MnO_2_ NFs. **Fig. S6**. Effects of (a) pH and (b) temperature on laccase-like activity of H–Mn–Cu NFs. **Fig. S7**. Comparison of stabilities between H–Mn–Cu NFs and free laccase in the following ranges: (a) pH, (b) temperature, (c) ionic concentration (NaCl), and (d) incubation period under shaking conditions. **Fig. S8**. Steady-state kinetic assays of (a) H–Mn–Cu NFs and (c) free laccase for epinephrine and their corresponding Lineweaver–Burk plots (b and d). **Fig. S9**. Comparison of laccase-like dopamine-detecting activity of H-Mn-Cu NFs with those of control samples. **Fig. S10**. Dose–response curves and corresponding linear calibration plots for determining (a, b) dopamine and (c, d) epinephrine using a well plate-based assay with free laccase. **Fig. S11**. MALDI-TOF mass spectrometry for H-Mn-Cu NFs-mediated degradation of (a) CV, (b) NR, and (c) RB at different incubation time of 0 h, 24 h, and 48 h. **Fig. S12**. Comparison of color signals of the H–Mn–Cu NF-incorporated paper microfluidic device for detecting both dopamine and epinephrine with those of controls [MnO_2_ NFs, Cu_3_(PO_4_)_2_ precipitates, and free laccase]. **Fig. S13**. Optimization of the wax barrier thickness of the paper microfluidic device to prevent flow leakage. (a) Paper devices after printing, (b) paper devices after wax melting, and (c) paper devices after adding a colored reagent (rhodamine B dye). The barrier thicknesses from top-left to down-right were 0.05, 0.1, 0.2, 0.3, 0.4, 0.5, 0.6, 0.7, 0.8, and 0.9 mm. Among these, 0.7 mm was used in this study. **Fig. S14**. Optimization of the reaction time for dopamine and epinephrine detection by H–Mn–Cu NF-incorporated paper microfluidic devices. Paper devices (a) before adding 4-AP, (b) after adding 4-AP, and for detecting dopamine and epinephrine (20 µM) during the reaction for (c) 0 min, (d) 2 min, (e) 5 min, (f) 10 min, (g) 15 min, (h) 20 min, (i) 25 min, and (j) 30 min. Among these, a 10 min reaction was used in this study. **Fig. S15**. Optimization of the employed concentrations of (a) 4-AP and (b) H–Mn–Cu NFs to prepare the H–Mn–Cu NF-incorporated paper microfluidic device for dopamine detection (20 µM). Consequently, 5 mg mL-1 4-AP and 10 mg mL-1 H–Mn–Cu NFs were used in this study. **Fig. S16** Long-term storage stability of the H–Mn–Cu NF-incorporated paper microfluidic device at RT. **Table S1**. Comparison of the linear range and LOD values of H-Mn-Cu NFs with those of recent colorimetric assays for dopamine and epinephrine. **Table S2**. Detection precision of the H–Mn–Cu NF-based paper microfluidic device for the determination of dopamine and epinephrine levels in spiked human serum samples.

## Data Availability

All data generated or analyzed during this study are included in this published article and its additional file.
