# Supplementary material for: Laccase-mimicking Mn–Cu hybrid nanoflowers for paper-based visual detection of phenolic neurotransmitters and rapid degradation of dyes
Source: J Nanobiotechnology. 2022 Aug 2;20:358. doi: 10.1186/s12951-022-01560-0 (PMC9344716; doi:10.1186/s12951-022-01560-0)
Supplement: Supplementary file 1 — Additional file 1: Fig. S1. SEM images of H–Mn–Cu NFs after incubation for (a) 0, (b) 12, (c) 24, (d) 48, and (e) 72 h. Insets represent high-resolution SEM images. Fig. S2. Elemental mapping of H–Mn–Cu NFs. Fig. S3. (a) XRD pattern and (b) FT-IR spectra of MnO2 NFs, amine-functionalized (APTES)-MnO2 NFs, Cu3(PO4)2 precipitates, and H–Mn–Cu NFs. (c) BET surface areas of MnO2 NFs, APTES-MnO2 NFs, and H–Mn–Cu NFs. Fig. S4. XPS full scan spectrum of H-Mn-Cu NFs. Fig. S5. High-resolution XPS spectra of Cu for (a) H-Mn-Cu NFs and (b) Cu3(PO4)2 precipitates, and Mn for (c) H-Mn-Cu NFs and (d) bare MnO2 NFs. Fig. S6. Effects of (a) pH and (b) temperature on laccase-like activity of H–Mn–Cu NFs. Fig. S7. Comparison of stabilities between H–Mn–Cu NFs and free laccase in the following ranges: (a) pH, (b) temperature, (c) ionic concentration (NaCl), and (d) incubation period under shaking conditions. Fig. S8. Steady-state kinetic assays of (a) H–Mn–Cu NFs and (c) free laccase for epinephrine and their corresponding Lineweaver–Burk plots (b and d). Fig. S9. Comparison of laccase-like dopamine-detecting activity of H-Mn-Cu NFs with those of control samples. Fig. S10. Dose–response curves and corresponding linear calibration plots for determining (a, b) dopamine and (c, d) epinephrine using a well plate-based assay with free laccase. Fig. S11. MALDI-TOF mass spectrometry for H-Mn-Cu NFs-mediated degradation of (a) CV, (b) NR, and (c) RB at different incubation time of 0 h, 24 h, and 48 h. Fig. S12. Comparison of color signals of the H–Mn–Cu NF-incorporated paper microfluidic device for detecting both dopamine and epinephrine with those of controls [MnO2 NFs, Cu3(PO4)2 precipitates, and free laccase]. Fig. S13. Optimization of the wax barrier thickness of the paper microfluidic device to prevent flow leakage. (a) Paper devices after printing, (b) paper devices after wax melting, and (c) paper devices after adding a colored reagent (rhodamine B dye). The barrier thicknesses from to [file 12951_2022_1560_MOESM1_ESM.docx]

Additional file 1

Supplementary Information

**Laccase-mimicking Mn–Cu hybrid nanoflowers for paper-based visual detection of** **phenolic neurotransmitters and rapid degradation of dyes**

Thao Nguyen Le^†^, Xuan Ai Le^†^, Tai Duc Tran, Kang Jin Lee, and Moon Il Kim*

Department of BioNano Technology, Gachon University, 1342 Seongnamdae-ro, Sujeong-gu, Seongnam, Gyeonggi 13120, Republic of Korea

*Correspondence: Tel. 82-31-750-8563; fax. 82-31-750-4748; email: moonil@gachon.ac.kr (M. I. K.)

^†^These authors contributed equally to this work.


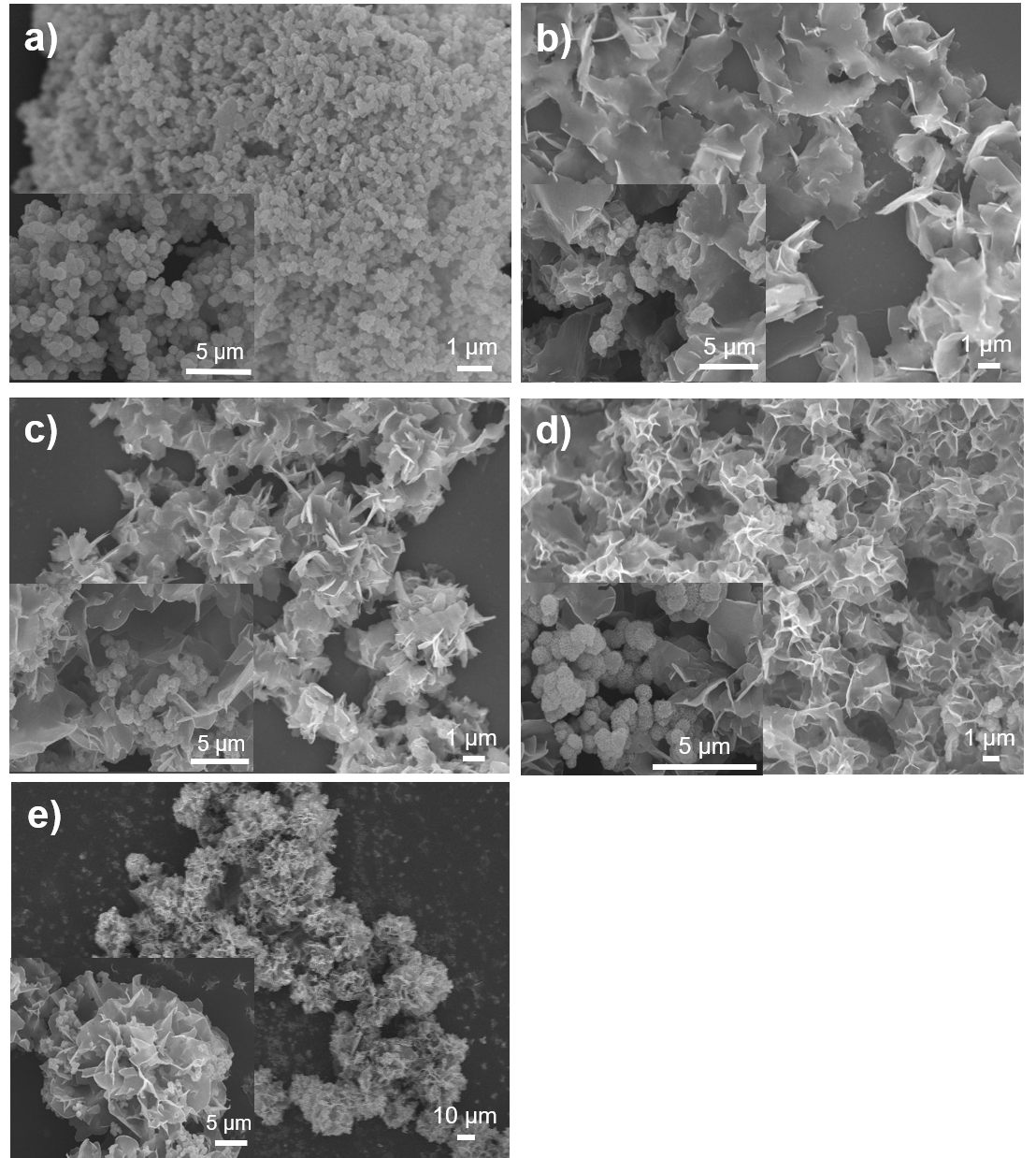


**Fig. S1** SEM images of H–Mn–Cu NFs after incubation for (a) 0, (b) 12, (c) 24, (d) 48, and (e) 72 h. Insets represent high-resolution SEM images.


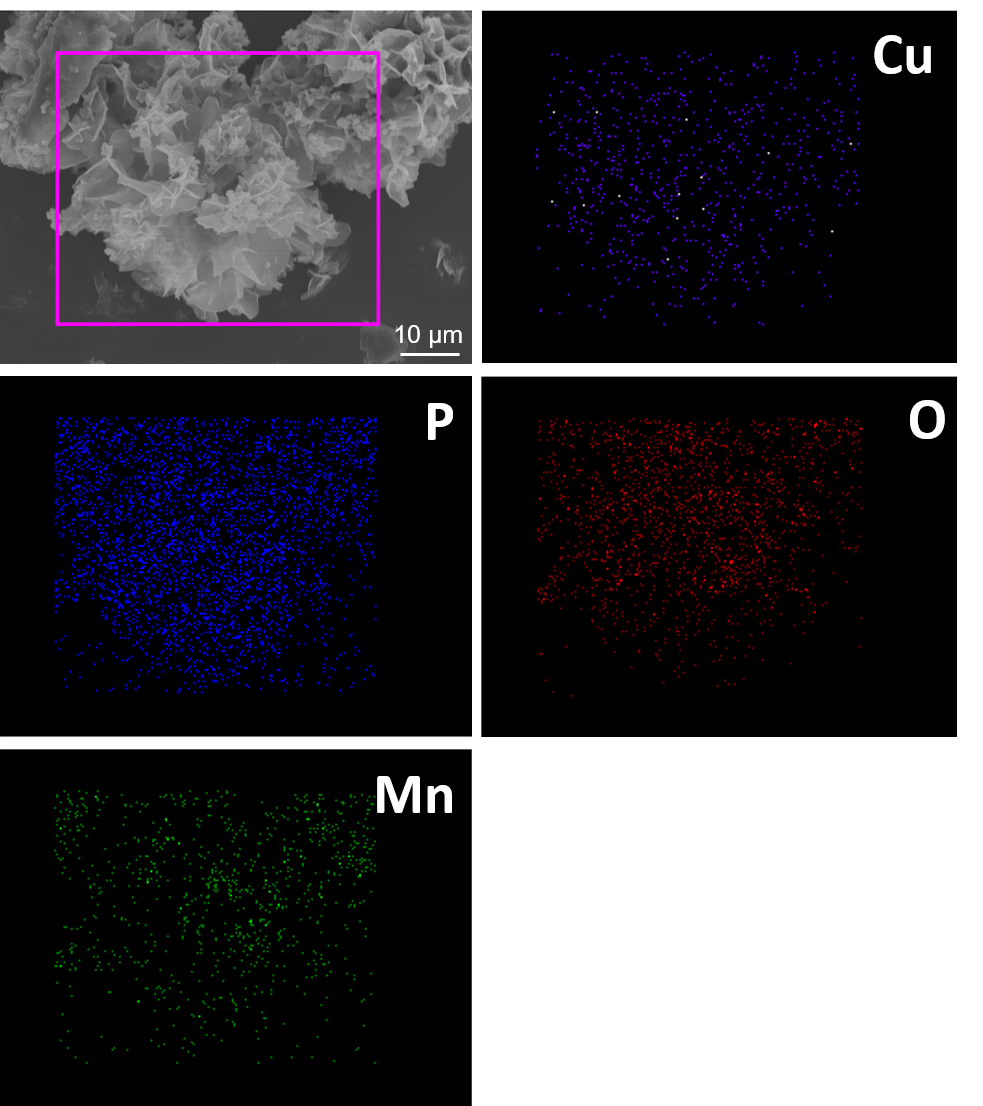


**Fig. S2** Elemental mapping of H–Mn–Cu NFs.


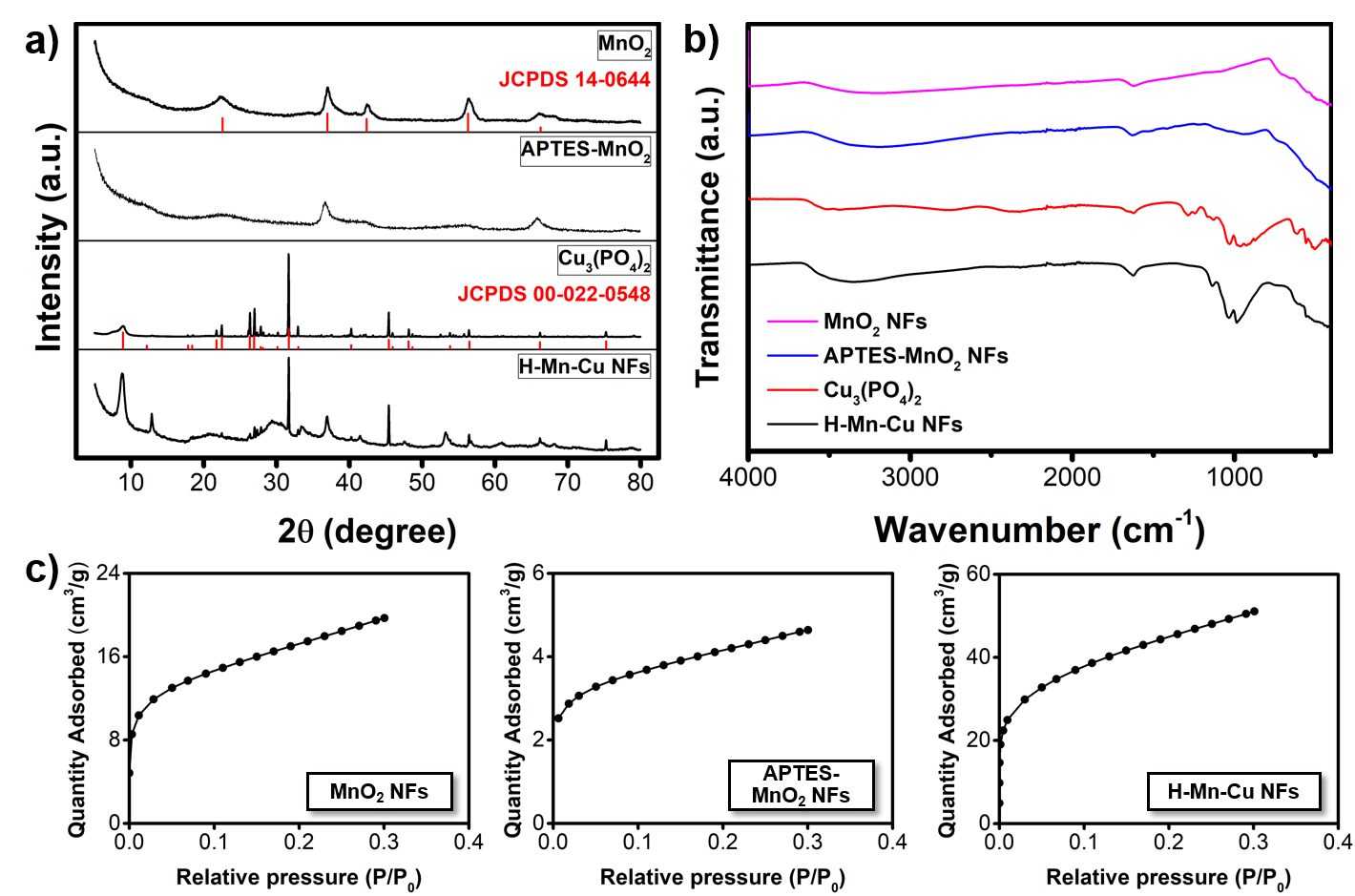


**Fig. S3** (a) XRD pattern and (b) FT-IR spectra of MnO_2_ NFs, amine-functionalized (APTES)-MnO_2_ NFs, Cu_3_(PO_4_)_2_ precipitates, and H–Mn–Cu NFs. (c) BET surface areas of MnO_2_ NFs, APTES-MnO_2_ NFs, and H–Mn–Cu NFs.


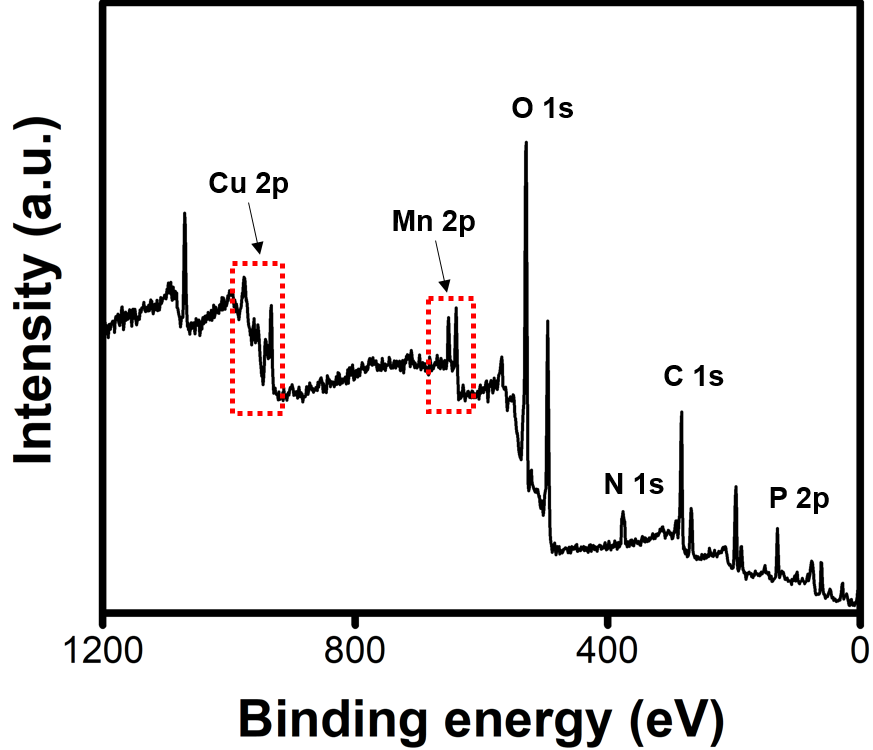


**Fig. S4** XPS full scan spectrum of H-Mn-Cu NFs.


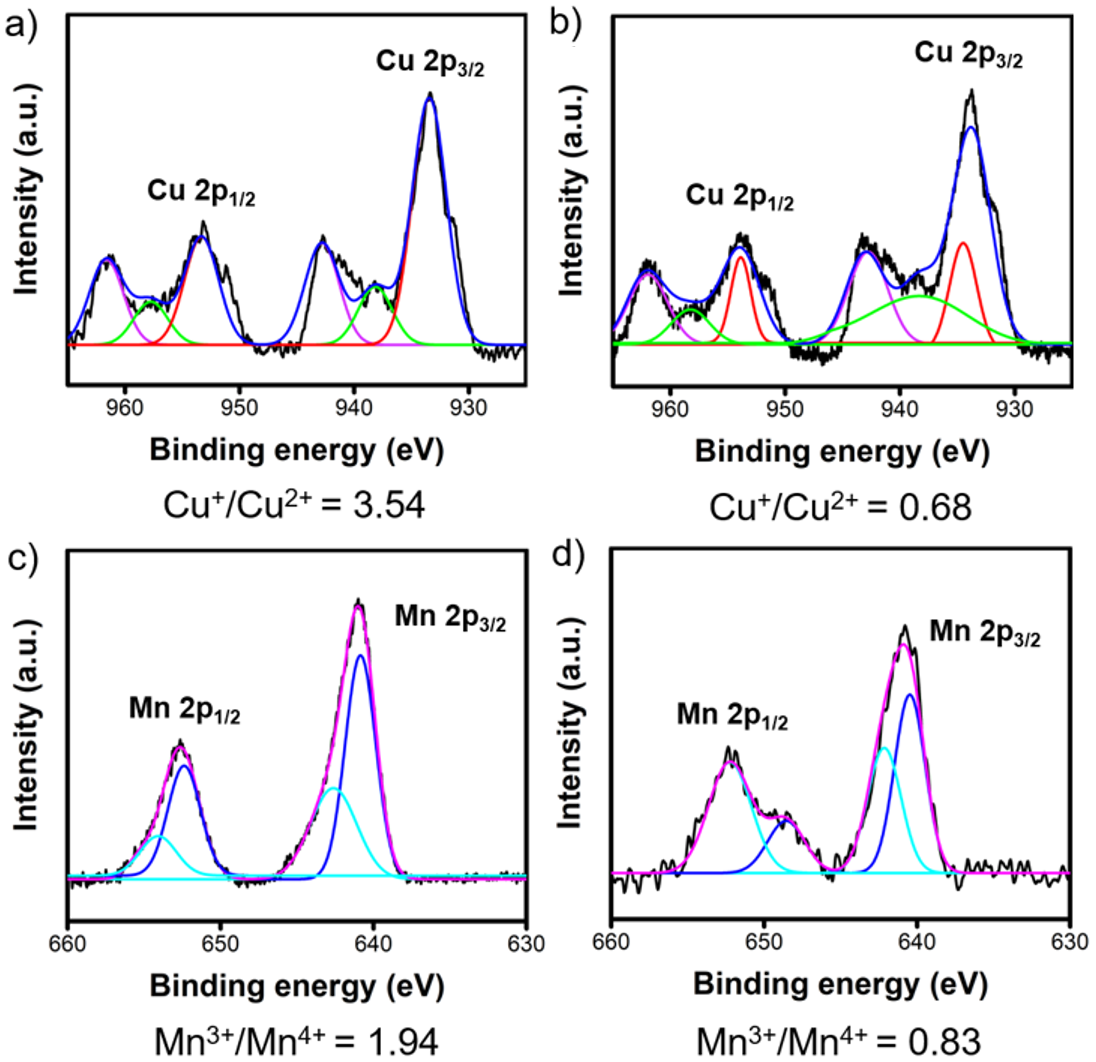


**Fig. S5** High-resolution XPS spectra of Cu for a) H-Mn-Cu NFs and b) Cu_3_(PO_4_)_2_ precipitates, and Mn for c) H-Mn-Cu NFs and d) bare MnO_2_ NFs. In a) and b), the red and green lines correspond to Cu^+^ and Cu^2+^, respectively. In c) and d), the blue and cyan lines represent Mn^3+^ and Mn^4+^, respectively.


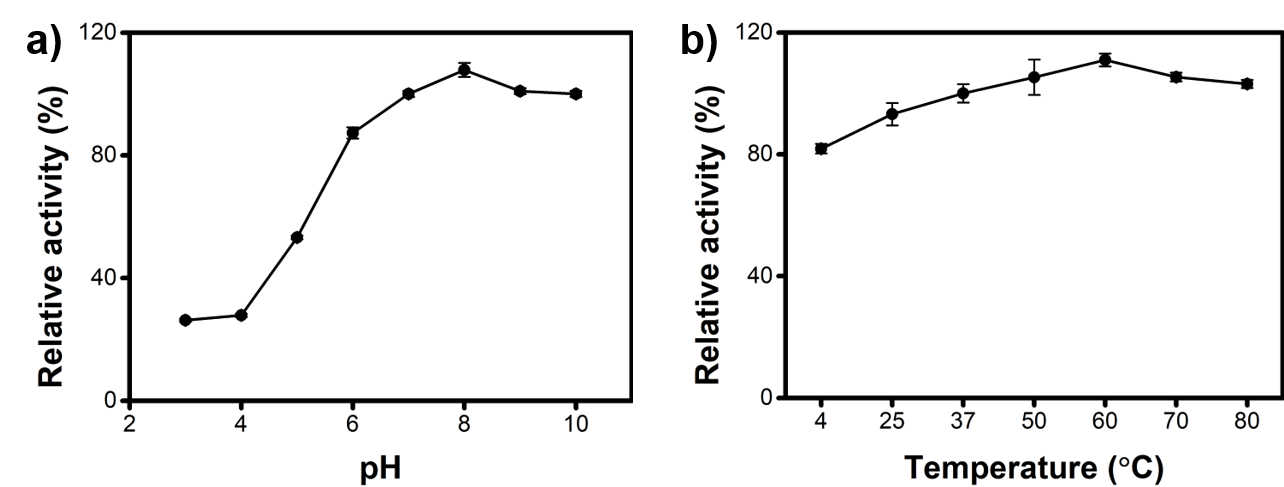


**Fig. S6** Effects of (a) pH and (b) temperature on laccase-like activity of H–Mn–Cu NFs.


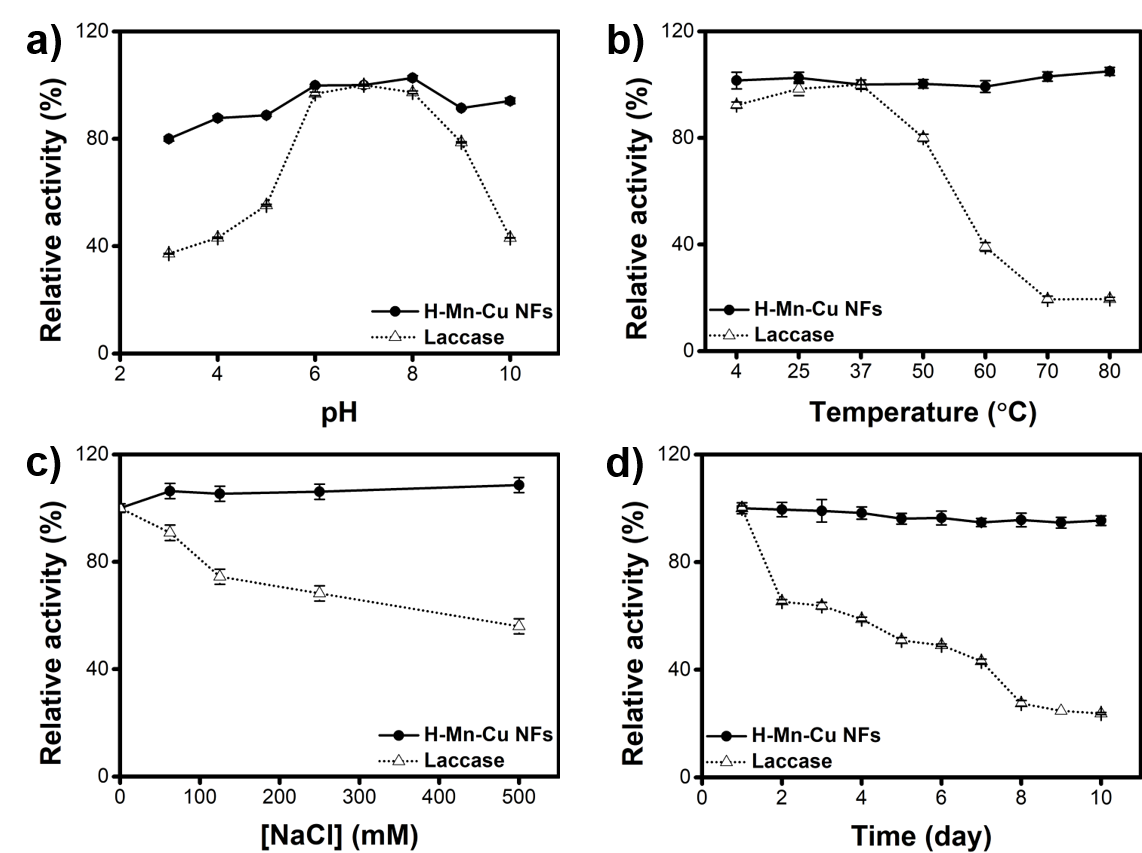


**Fig. S7** Comparison of stabilities between H–Mn–Cu NFs and free laccase in the following ranges: (a) pH, (b) temperature, (c) ionic concentration (NaCl), and (d) incubation period under shaking conditions.


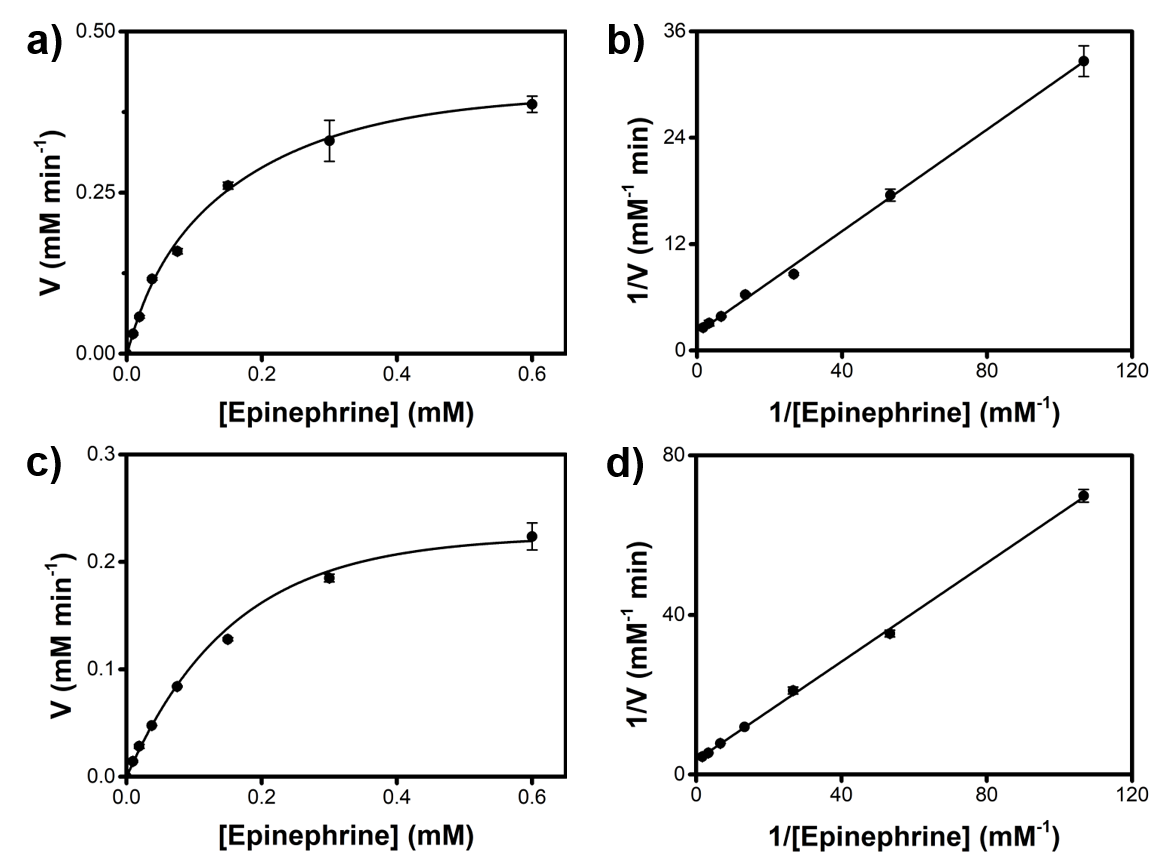


**Fig. S8** Steady-state kinetic assays of (a) H–Mn–Cu NFs and (c) free laccase for epinephrine and their corresponding Lineweaver–Burk plots (b and d).


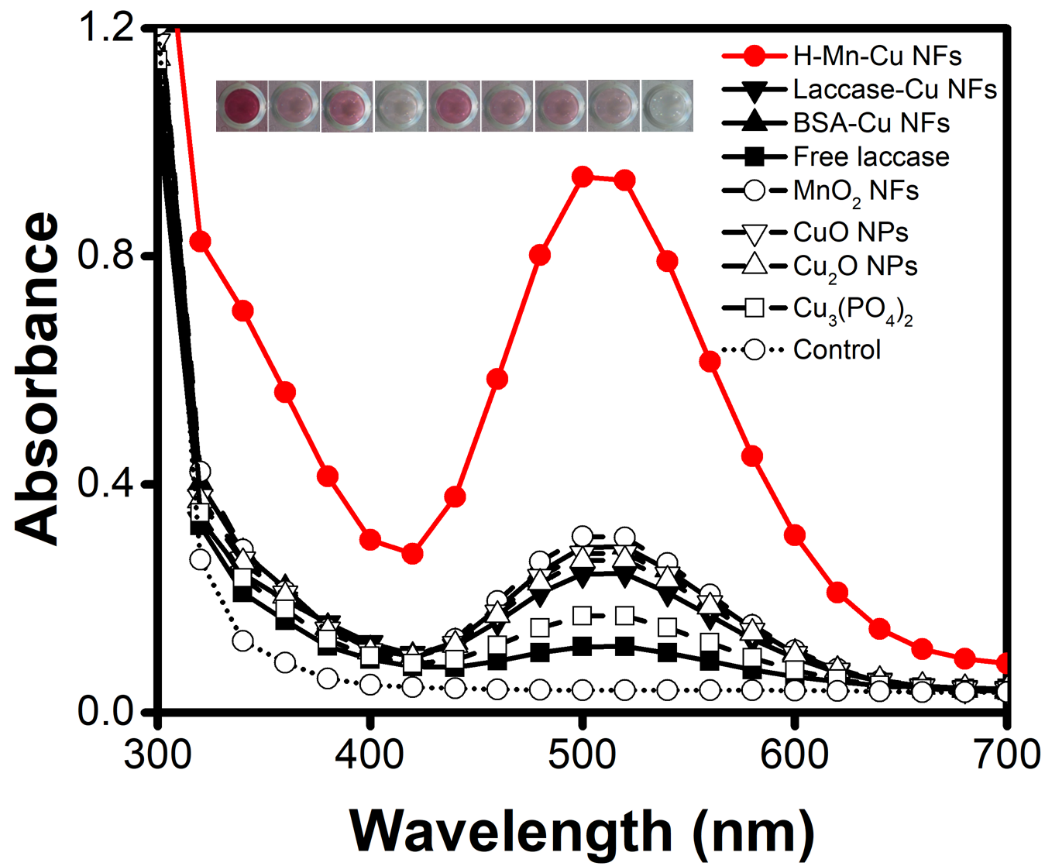


**Fig. S9** Comparison of laccase-like dopamine-detecting activity of H-Mn-Cu NFs with those of control samples. As control samples, laccase-Cu NFs and BSA-Cu NFs were prepared with the same self-assembly method used for H-Mn-Cu NFs, except using free laccase and BSA as organic components instead of amine-functionalized MnO_2_ NFs. CuO NPs (< 50 nm in diameter) and Cu_2_O NPs (< 350 nm in diameter) were purchased from Sigma-Aldrich. Dopamine-detecting laccase-like activity was evaluated by mixing laccase-like catalysts (0.1 mg mL^-1^) with dopamine (0.1 mg mL^-1^) and 4-AP (0.1 mg mL^-1^) in MEB buffer (50 mM, pH 6.8), followed by incubation for 40 min at RT. After the reaction, the mixture was centrifuged at 10,000 rpm for 2 min, and the absorbance spectra of the supernatant was recorded.


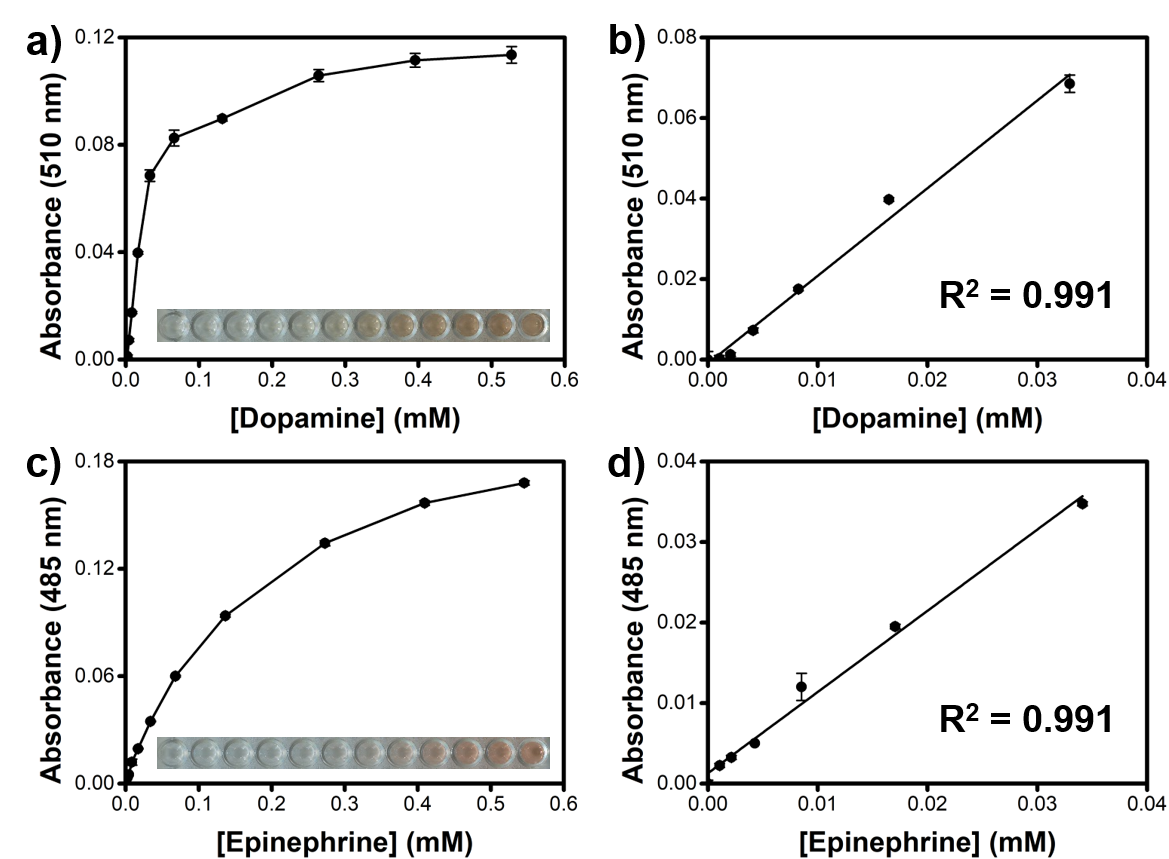


**Fig. S10** Dose–response curves and corresponding linear calibration plots for determining (a, b) dopamine and (c, d) epinephrine using a well plate-based assay with free laccase.


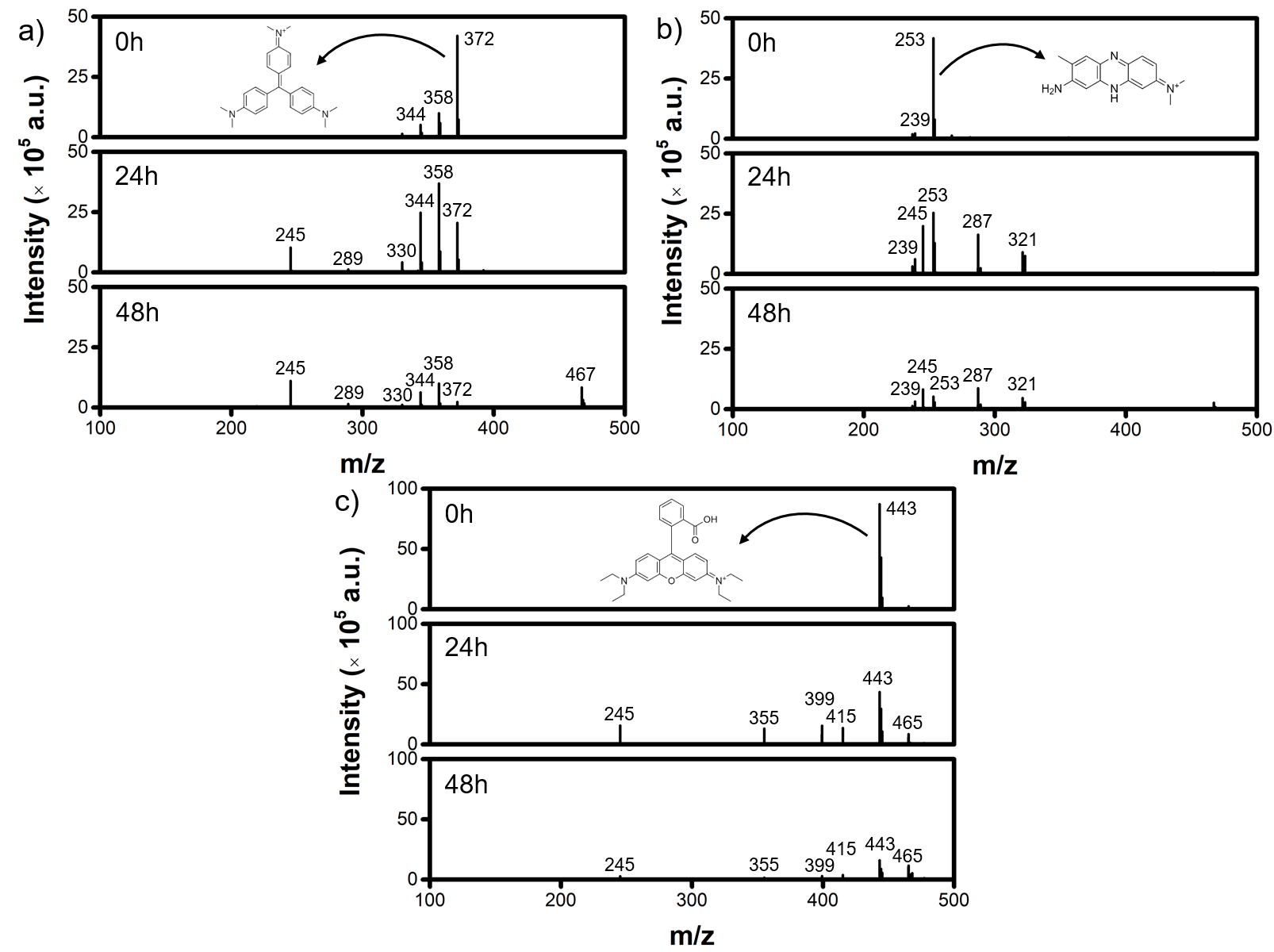


**Fig. S11** MALDI-TOF mass spectrometry for H-Mn-Cu NFs-mediated degradation of a) CV, b) NR, and c) RB at different incubation time of 0 h, 24 h, and 48 h.


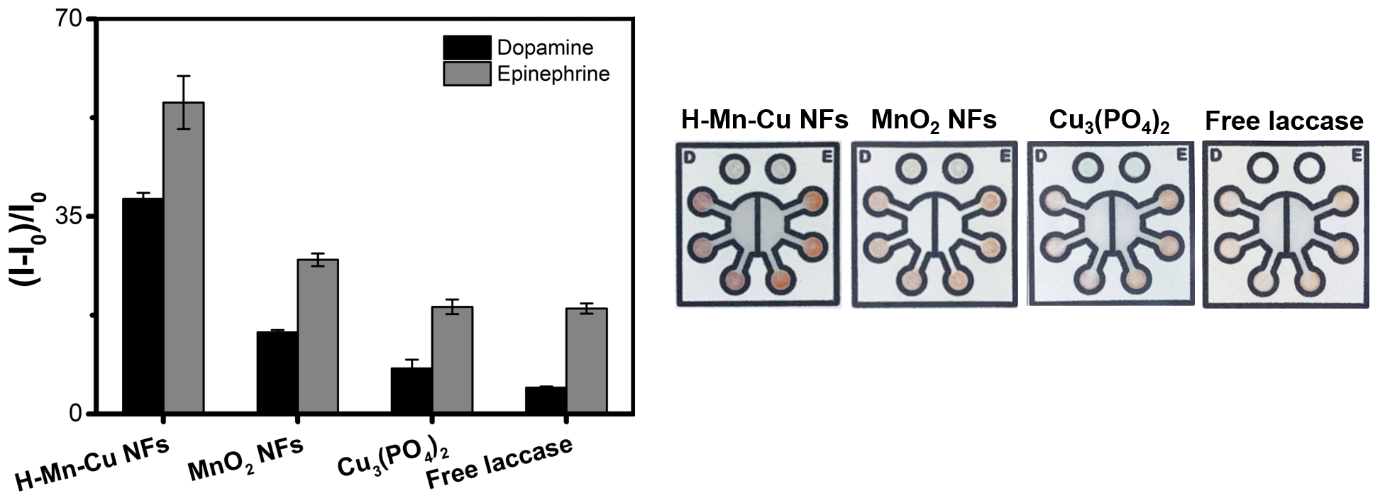


**Fig. S12** Comparison of color signals of the H–Mn–Cu NF-incorporated paper microfluidic device for detecting both dopamine and epinephrine with those of controls [MnO_2_ NFs, Cu_3_(PO_4_)_2_ precipitates, and free laccase].


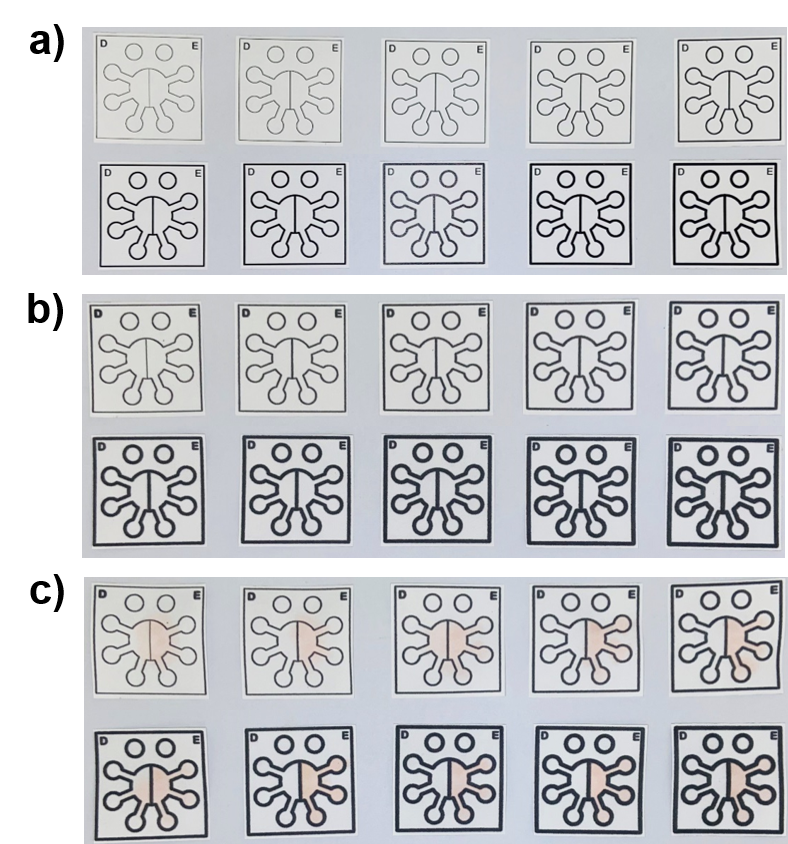


**Fig. S13** Optimization of the wax barrier thickness of the paper microfluidic device to prevent flow leakage. (a) Paper devices after printing, (b) paper devices after wax melting, and (c) paper devices after adding a colored reagent (rhodamine B dye). The barrier thicknesses from top-left to down-right were 0.05, 0.1, 0.2, 0.3, 0.4, 0.5, 0.6, 0.7, 0.8, and 0.9 mm. Among these, 0.7 mm was used in this study.


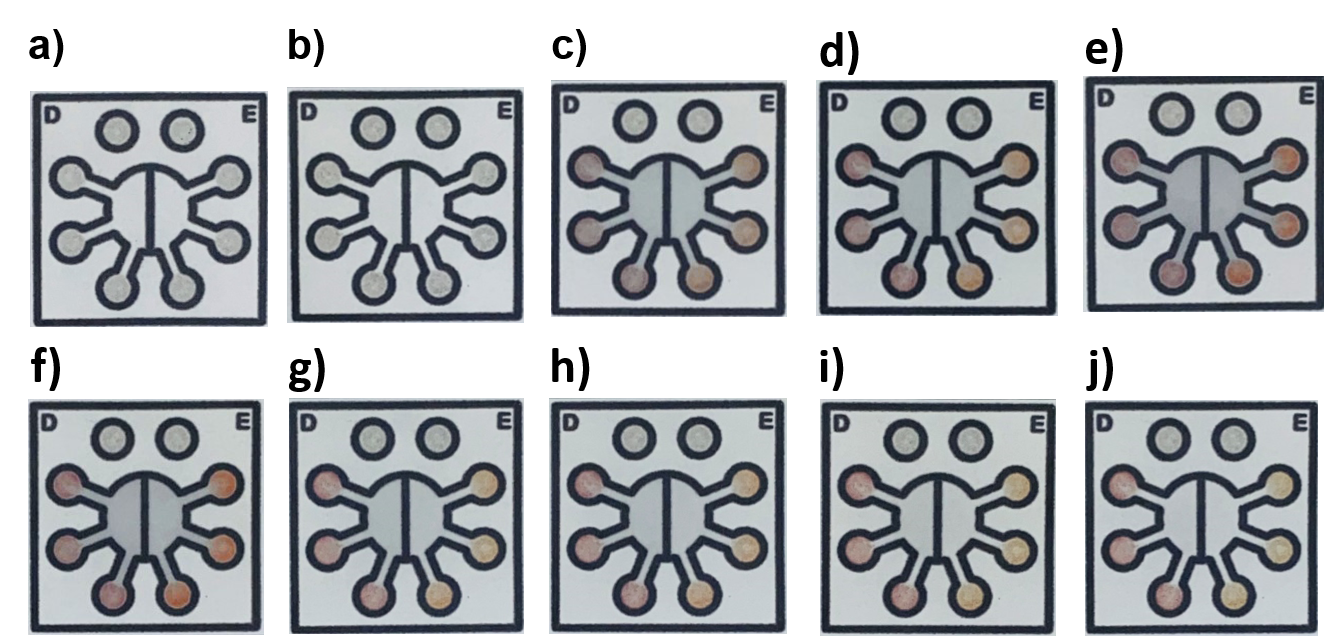


**Fig. S14** Optimization of the reaction time for dopamine and epinephrine detection by H–Mn–Cu NF-incorporated paper microfluidic devices. Paper devices (a) before adding 4-AP, (b) after adding 4-AP, and for detecting dopamine and epinephrine (20 μM) during the reaction for (c) 0 min, (d) 2 min, (e) 5 min, (f) 10 min, (g) 15 min, (h) 20 min, (i) 25 min, and (j) 30 min. Among these, a 10 min reaction was used in this study.


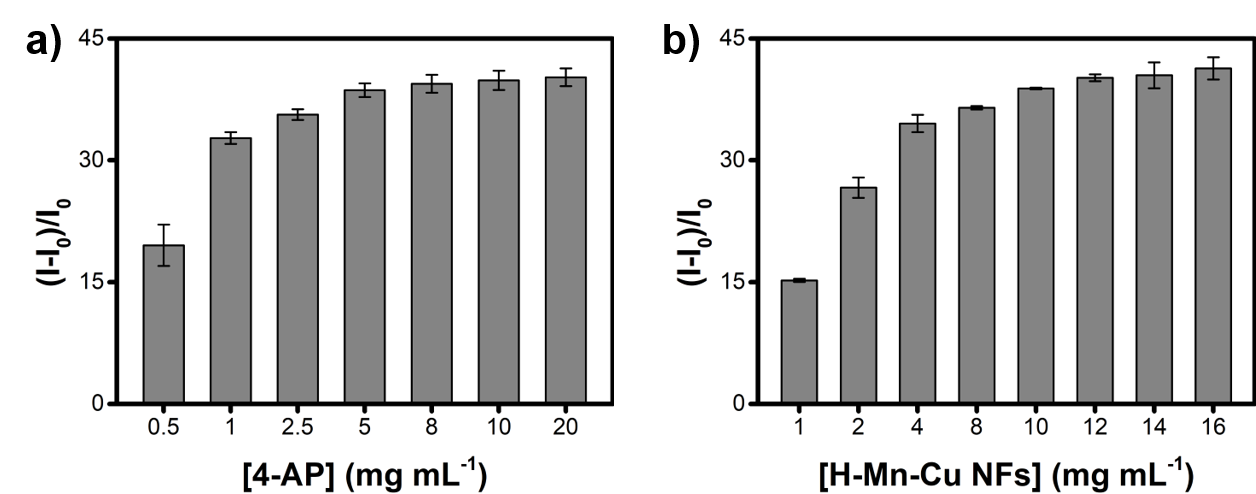


**Fig. S15** Optimization of the employed concentrations of (a) 4-AP and (b) H–Mn–Cu NFs to prepare the H–Mn–Cu NF-incorporated paper microfluidic device for dopamine detection (20 μM). Consequently, 5 mg mL^-1^ 4-AP and 10 mg mL^-1^ H–Mn–Cu NFs were used in this study.


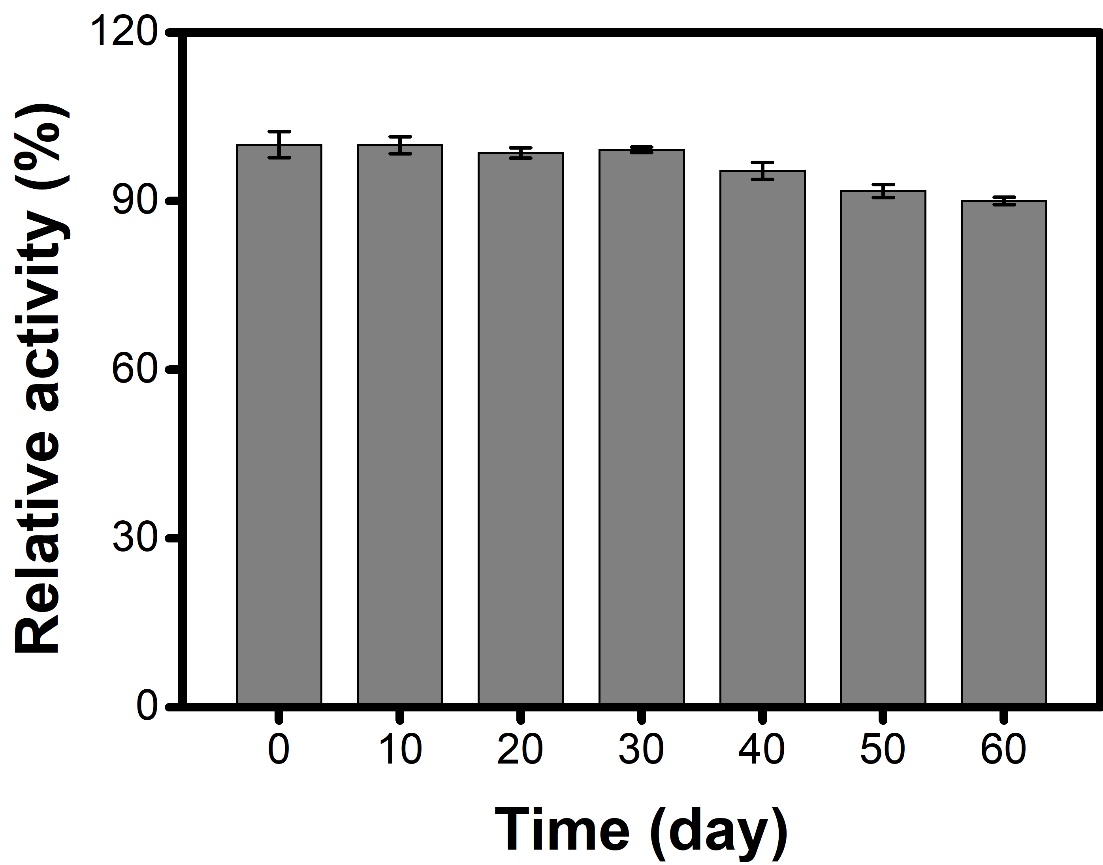


**Fig. S16** Long-term storage stability of the H–Mn–Cu NF-incorporated paper microfluidic device at RT.

**Table S1** Comparison of the linear range and LOD values of H-Mn-Cu NFs with those of recent colorimetric assays for dopamine and epinephrine.

| **Neurotransmitters** | **Materials** | **Linear range (µM)** | **LOD (nM)** | **References** |
| --- | --- | --- | --- | --- |
| Dopamine | IL capped Ag NPs^a^ | 0.01 – 3.6 | 118 | [1] |
|  | Au NPs | 0.5 – 10.0 | 200 | [2] |
|  | CuS/rGO^b^ | 2.0 – 100.0 | 430 | [3] |
|  | DNA-Cu NFs^c^  (paper device) | 0 – 131.8 | 23.7 × 10^3^ | [4] |
|  | H-Mn-Cu NFs (solution) | 1.0 – 33.0 | 84.9 | This work |
|  | H-Mn-Cu NFs  (paper device) | 0.5 – 5.0 | 54.0 | This work |
| Epinephrine | DNA-Cu NFs | 0 – 136.5 | 1.6 × 10^3^ | [4] |
|  | La-HMFs^d^ | 1.0 – 400.0 | 600 | [5] |
|  | CuO nanorods | 0.6 – 18.0 | 310 | [6] |
|  | H-Mn-Cu NFs (solution) | 1.1 – 34.1 | 100.2 | This work |
|  | H-Mn-Cu NFs  (paper device) | 0.5 – 5.0 | 34.5 | This work |

^a^ Ionic liquid capped silver nanoparticles

^b^ Copper sulfide decorated reduced graphene oxide nanosheets

^c^ DNA-copper hybrid nanoflowers

^d^ Laccase-mineral hybrid microflowers

**Table S2** Detection precision of the H–Mn–Cu NF-based paper microfluidic device for the determination of dopamine and epinephrine levels in spiked human serum samples.

| **Neurotransmitters** | **Added^a^ (μM)** | **Expected**  **(μM)** | **Measured^b^**  **(μM)** | **SD^c^** | **CV^d^ (%)** | **Recovery^e^ (%)** |
| --- | --- | --- | --- | --- | --- | --- |
| Dopamine | 5 | 5 | 5.1 | 0.18 | 3.60 | 101.4 |
|  | 10 | 10 | 10.4 | 0.49 | 4.72 | 103.9 |
|  | 20 | 20 | 19.6 | 0.55 | 2.79 | 98.1 |
| Epinephrine | 5 | 5 | 5.1 | 0.22 | 4.26 | 101.9 |
|  | 10 | 10 | 9.7 | 0.38 | 3.94 | 97.2 |
|  | 20 | 20 | 19.8 | 0.91 | 4.60 | 99.0 |

^a^ Concentration of dopamine and epinephrine in the original blood serum (Sigma-Aldrich) was lower than the detection limit of the method. However, considering the high patient levels of dopamine and epinephrine in human serum from diseases like Alzheimer, Schizophrenia, or Parkinsonism, the developed paper device can be utilized to discriminate between patients and normal, as well as to quantify patient levels [7,8].

^b^ Average value of six independent measurements.

^c^ Standard deviation (SD) of six successive measurements.

^d^ Coefficient of variation = (SD / average) × 100.

^e^ Recovery = (measured value/expected value) × 100.

**References**

1. Nishan U, Gul R, Muhammad N, Asad M, Rahim A, Shah M, et al. Colorimetric based sensing of dopamine using ionic liquid functionalized drug mediated silver nanostructures. Microchem J. 2020;159:105382.

2. Su H, Sun B, Chen L, Xu Z, Ai S. Colorimetric sensing of dopamine based on the aggregation of gold nanoparticles induced by copper ions. Anal Methods. 2012;4:3981-3986.

3. Dutta S, Ray C, Mallick S, Sarkar S, Sahoo R, Negishi Y, et al. A gel-based approach to design hierarchical CuS decorated reduced graphene oxide nanosheets for enhanced peroxidase-like activity leading to colorimetric detection of dopamine. J Phys Chem C. 2015;119:23790-23800.

4. Tran TD, Nguyen PT, Le TN, Kim MI. DNA-copper hybrid nanoflowers as efficient laccase mimics for colorimetric detection of phenolic compounds in paper microfluidic devices. Biosens Bioelectron. 2021;182:113187.

5. Zhang M, Zhang Y, Yang C, Ma C, Tang J. A smartphone-assisted portable biosensor using laccase-mineral hybrid microflowers for colorimetric determination of epinephrine. Talanta. 2021;224:121840.

6. Alizadeh N, Ghasemi S, Salimi A, Sham T-K, Hallaj R. CuO nanorods as a laccase mimicking enzyme for highly sensitive colorimetric and electrochemical dual biosensor: Application in living cell epinephrine analysis. Colloids Surf B: Biointerfaces. 2020;195:111228.

7. Wierzbicka E, Szultka-Młyńska M, Buszewski B, Sulka GD. Epinephrine sensing at nanostructured Au electrode and determination its oxidative metabolism. Sens Actuators B Chem. 2016;237:206-215.

8. Liu X, Liu J. Biosensors and sensors for dopamine detection. VIEW. 2021;2:20200102.
